# Supplementary material for: CK2 Inhibition and Antitumor Activity of 4,7-Dihydro-6-nitroazolo[1,5-a]pyrimidines
Source: Molecules. 2022 Aug 17;27(16):5239. doi: 10.3390/molecules27165239 (PMC9415015; doi:10.3390/molecules27165239)
Supplement: Supplementary file 1 [file molecules-27-05239-s001.zip › molecules-1832338-supplementary.pdf]

Supporting Information

for

# CK2 inhibition and antitumor activity of 4,7-dihydro-6-nitroazolo[1,5-a] pyrimidines

Daniil N. Lyapustin[1], Svetlana K. Kotovskaya[1], Ilya I. Butorin[1], Evgeny N. Ulomsky[1], Vladimir L. Rusinov[1], Denis A. Babkov[2], Alexander A. Pokhlebin[2], Alexander A. Spasov[2], Vsevolod V. Melekhin[1,3], Maria D. Tokhtueva[1], Anna V. Shcheglova[1,3] and Oleg G. Makeev[3]

[1]Ural Federal University named after the first President of Russia B.N. Eltsyn, Mira 19 st, 620002, Ekaterinburg, Russia

[2]Volgograd State Medicinal University, Pavshikh Bortsov 1 Sq, 400131, Volgograd, Russia

[3]Ural State Medical University, Repina 3 st, 620028, Ekaterinburg, Russia

E-mail: v.l.rusinov@urfu.ru

Table of Contents:

NMR Spectra of Compounds **4,5** S-2

# $^{1}H$, $^{13}C$ NMR spectra of the products 5,6

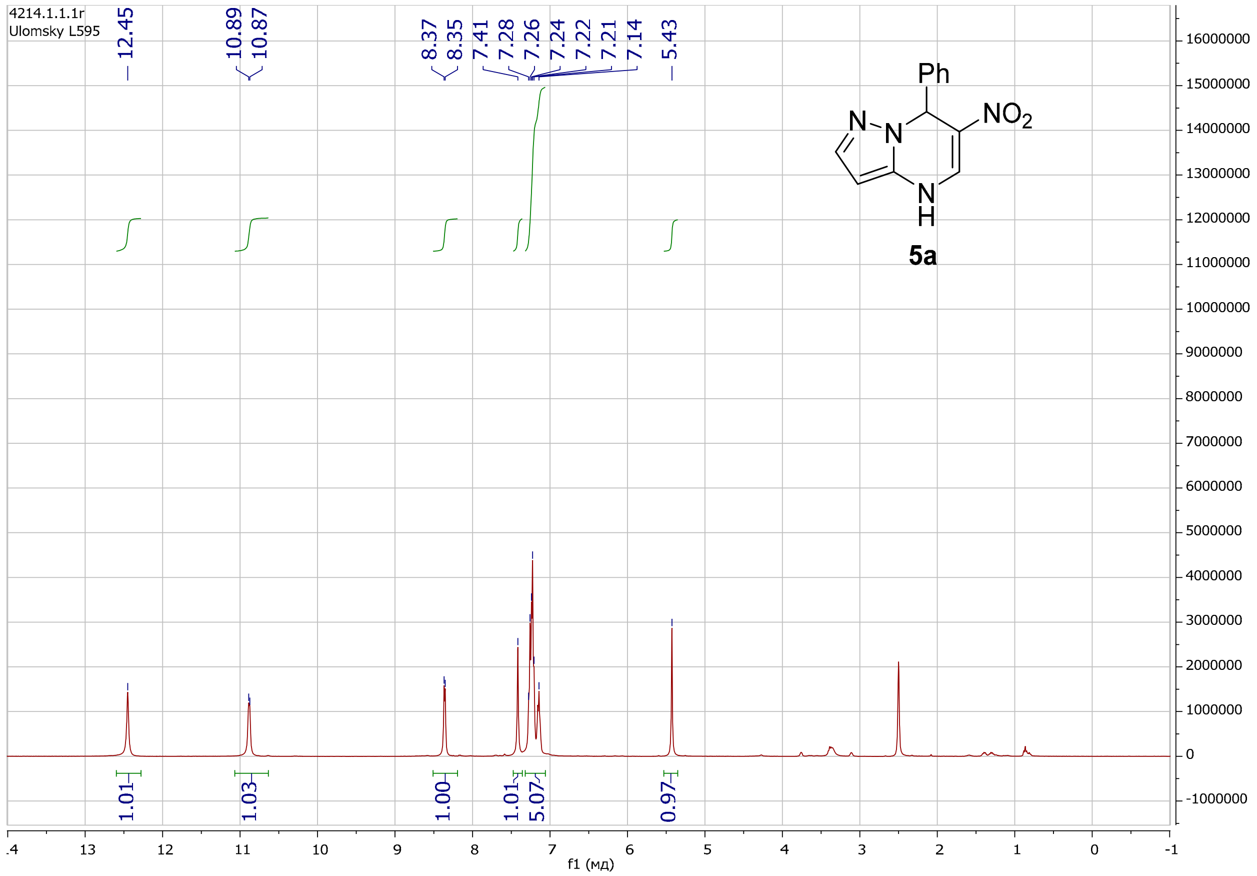


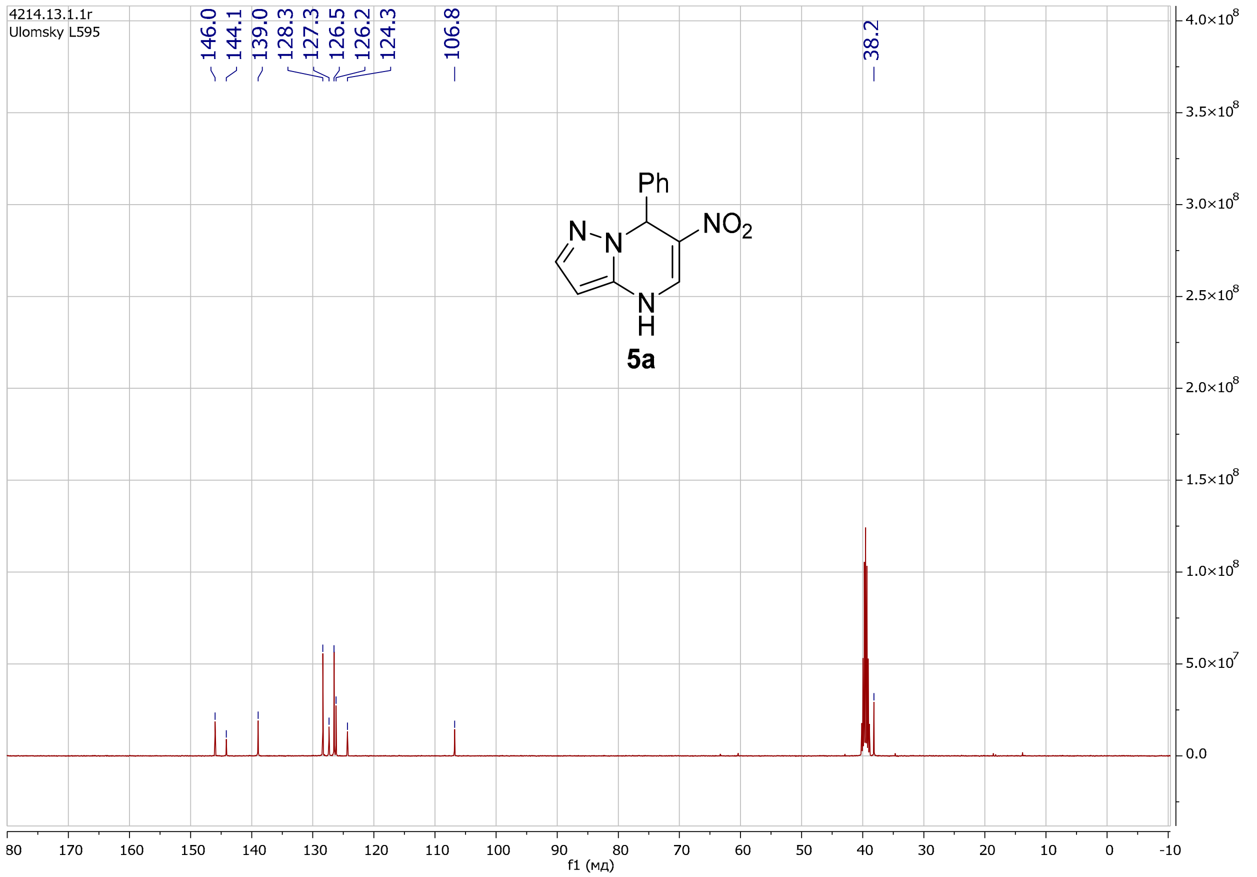

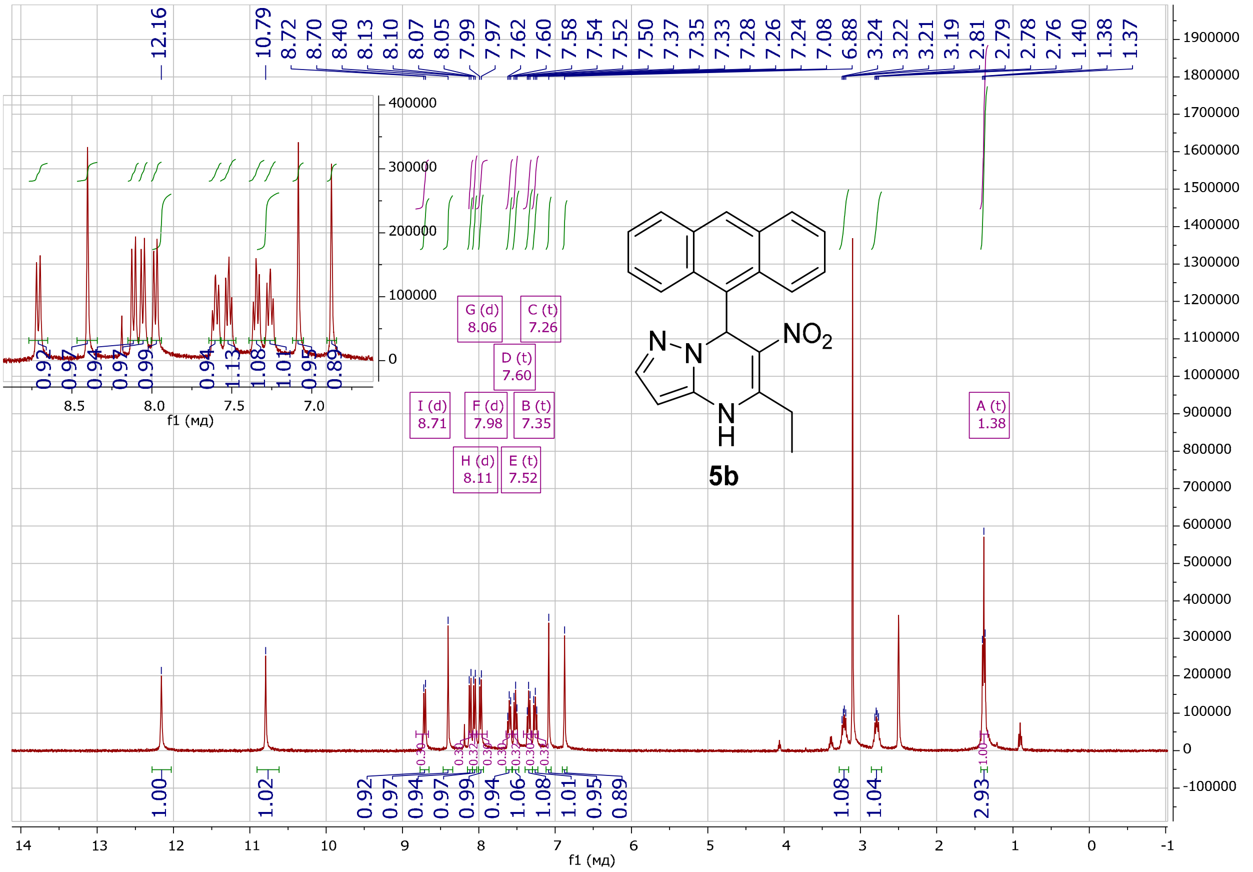

12.16
10.79
8.72
8.70
8.40
8.13
8.10
8.07
8.05
7.99
7.97
7.62
7.60
7.58
7.54
7.52
7.50
7.37
7.35
7.33
7.28
7.26
7.24
7.08
6.88
3.24
3.22
3.21
3.19
2.81
2.79
2.78
2.76
1.40
1.38
1.37
G (d) 8.06
C (t) 7.26
D (t) 7.60
I (d) 8.71
F (d) 7.98
B (t) 7.35
H (d) 8.11
E (t) 7.52
A (t) 1.38
NO2
N
N
N
H
5b
1.00
1.02
0.92
0.97
0.94
0.97
0.99
0.94
1.06
1.08
1.01
0.95
0.89
1.08
1.04
2.93
f1 (мд)


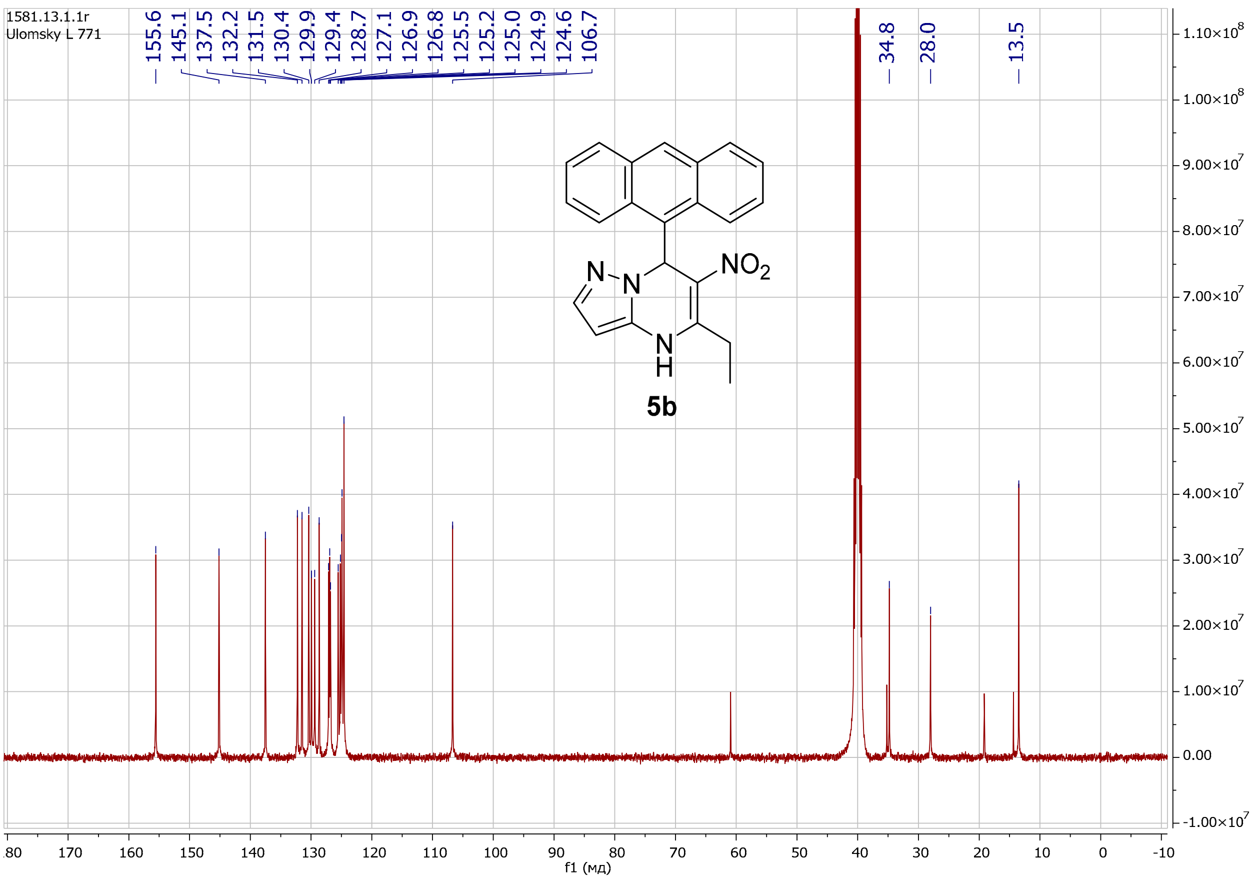

1581.13.1.1r
Ulomsky L 771
155.6
145.1
137.5
132.2
131.5
130.4
129.9
129.4
128.7
127.1
126.9
126.8
125.5
125.2
125.0
124.9
124.6
106.7
34.8
28.0
13.5
NO2
N
N
N
H
5b
f1 (мд)

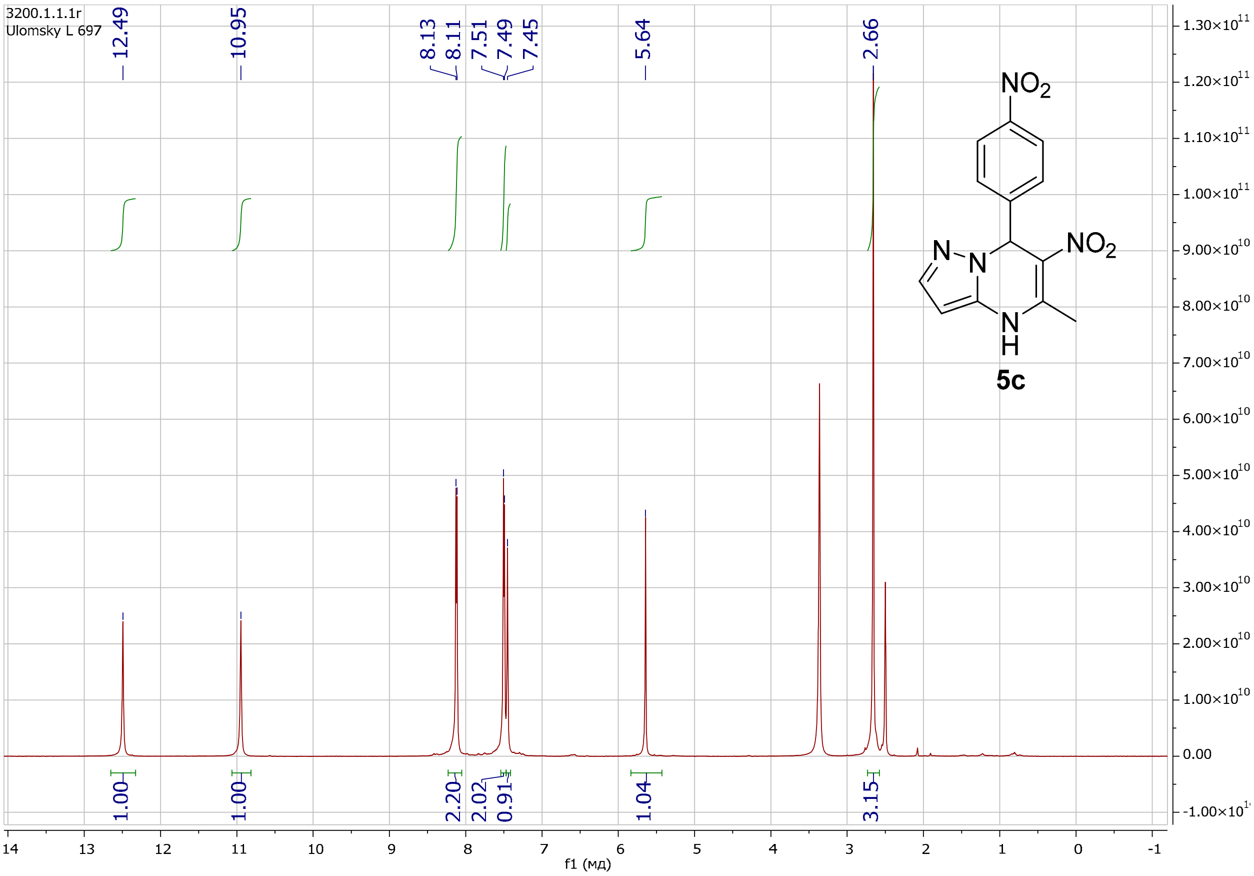
3200.1.1.1r
Ulomsky L 697
12.49
10.95
8.13
8.11
7.51
7.49
7.45
5.64
2.66
1.00
1.00
2.20
2.02
0.91
1.04
3.15
NO2
N
N
NO2
N
H
5c
f1 (мд)

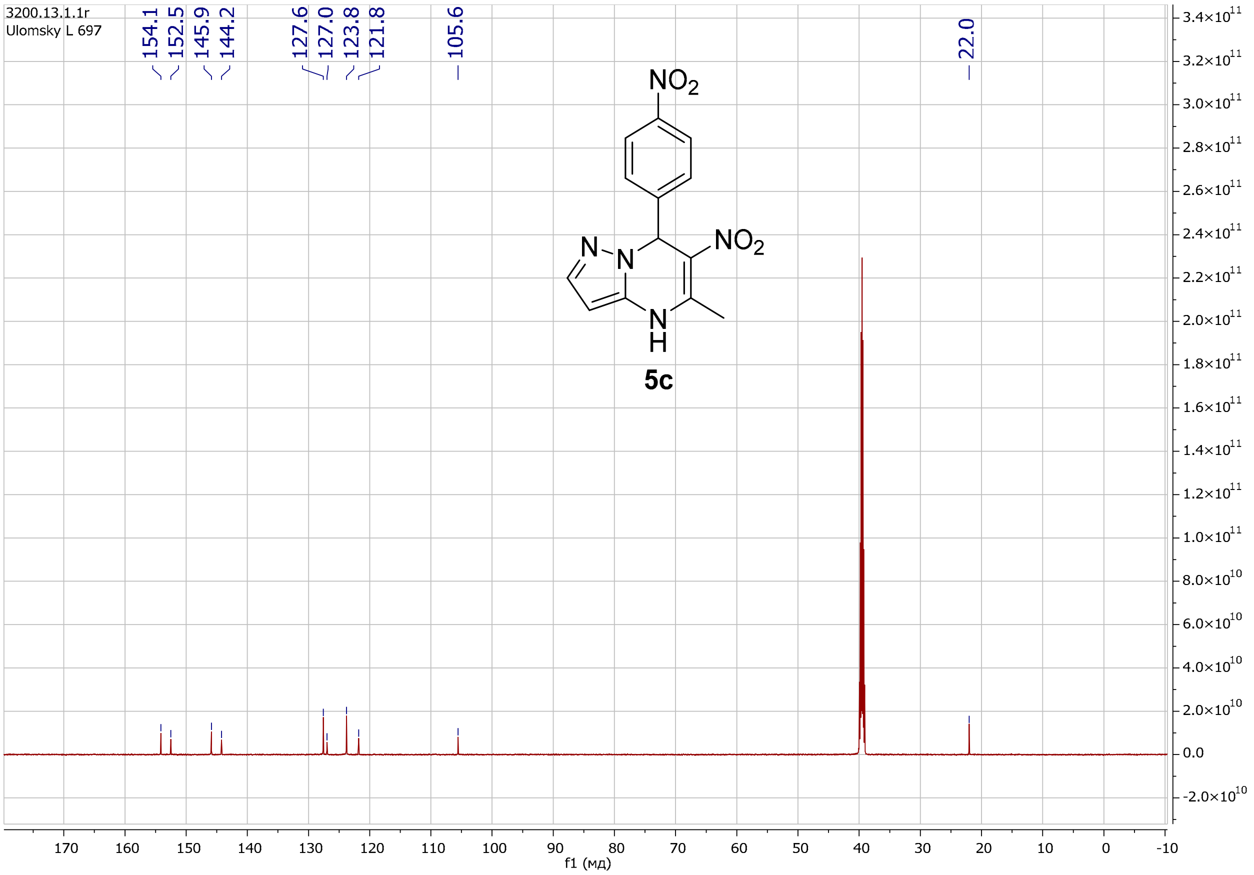
3200.13.1.1r
Ulomsky L 697
154.1
152.5
145.9
144.2
127.6
127.0
123.8
121.8
105.6
22.0
NO2
N
N
NO2
N
H
5c
f1 (мд)

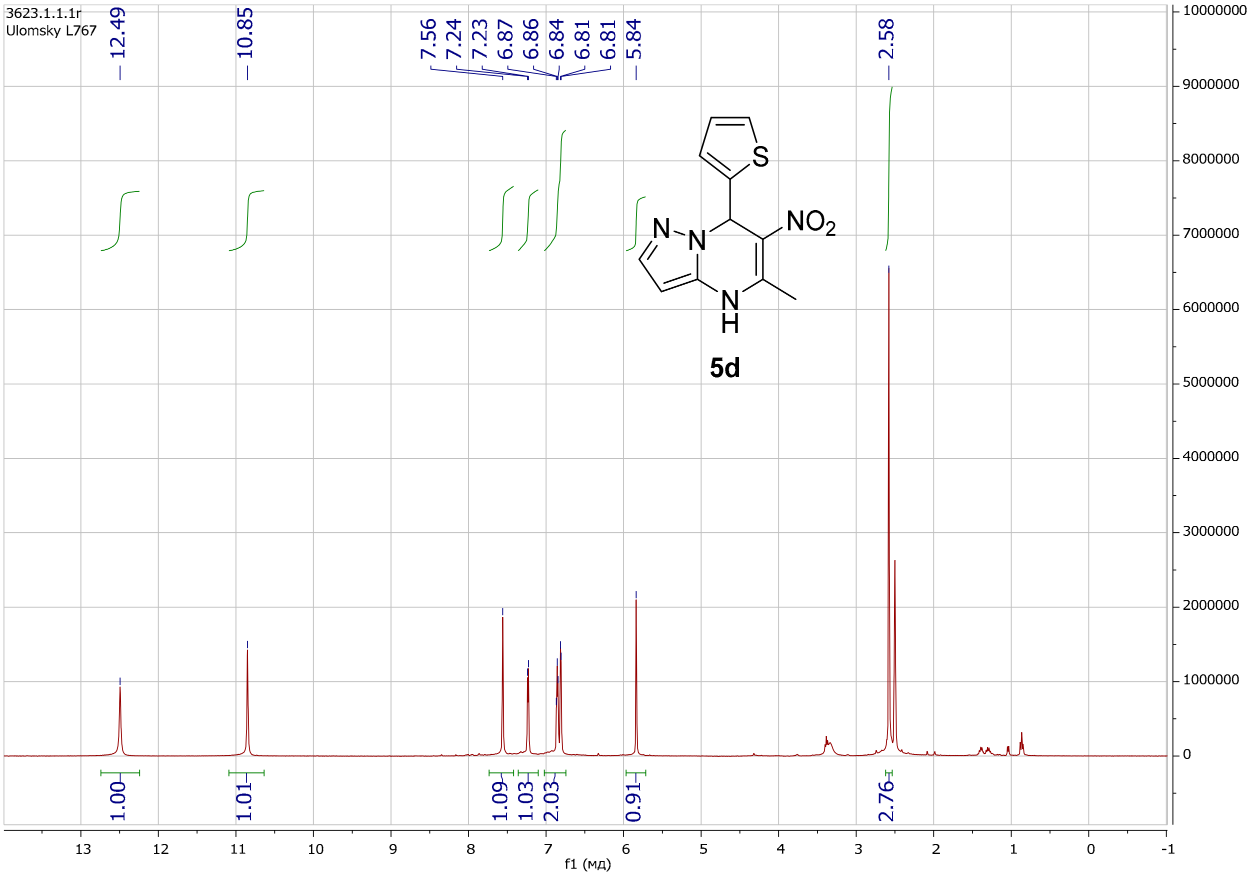
3623.1.1.1r
Ulomsky L767
12.49
10.85
7.56
7.24
7.23
6.87
6.86
6.84
6.81
6.81
5.84
2.58
NO2
N
N
N
H
S
5d
1.00
1.01
1.09
1.03
2.03
0.91
2.76
f1 (мд)

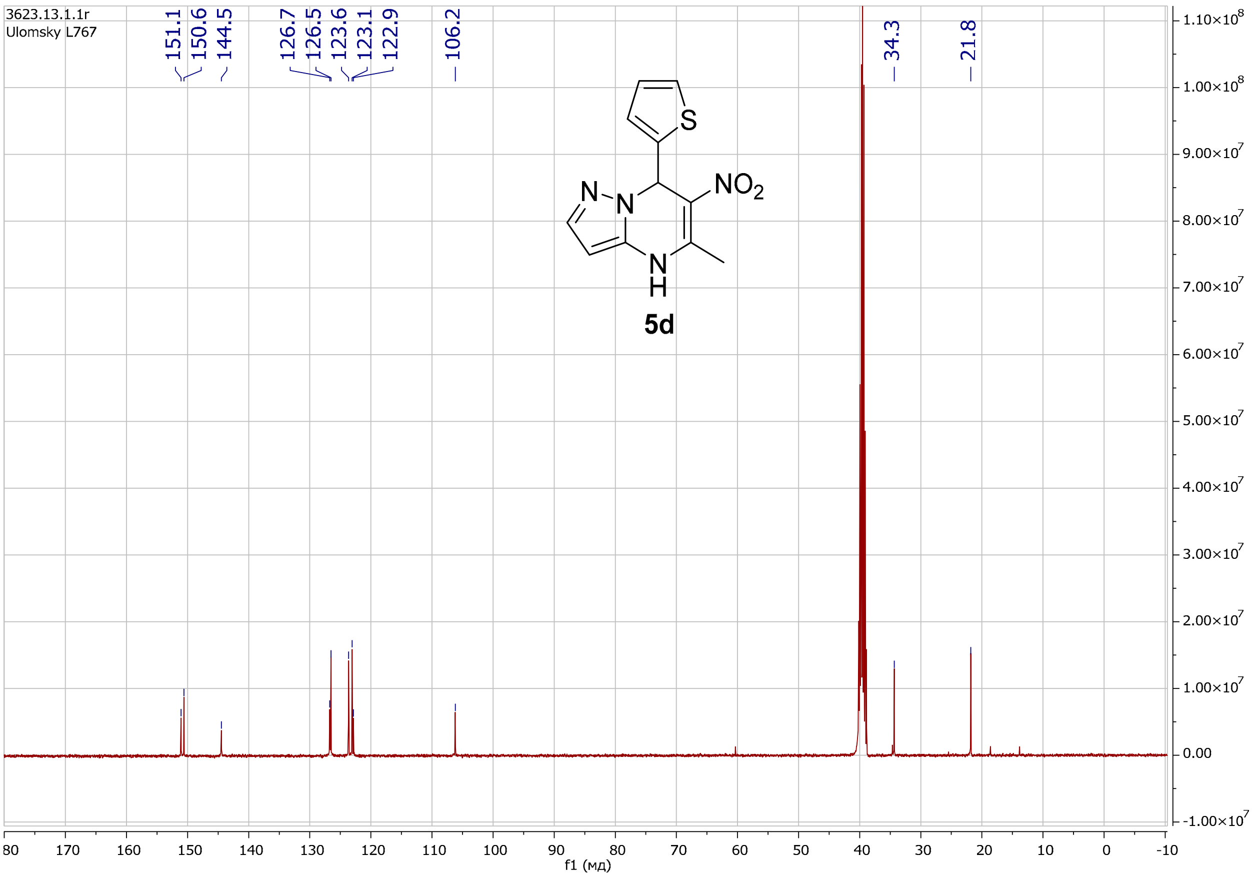
3623.13.1.1r
Ulomsky L767
151.1
150.6
144.5
126.7
126.5
123.6
123.1
122.9
106.2
34.3
21.8
NO2
N
N
N
H
S
5d
f1 (мд)

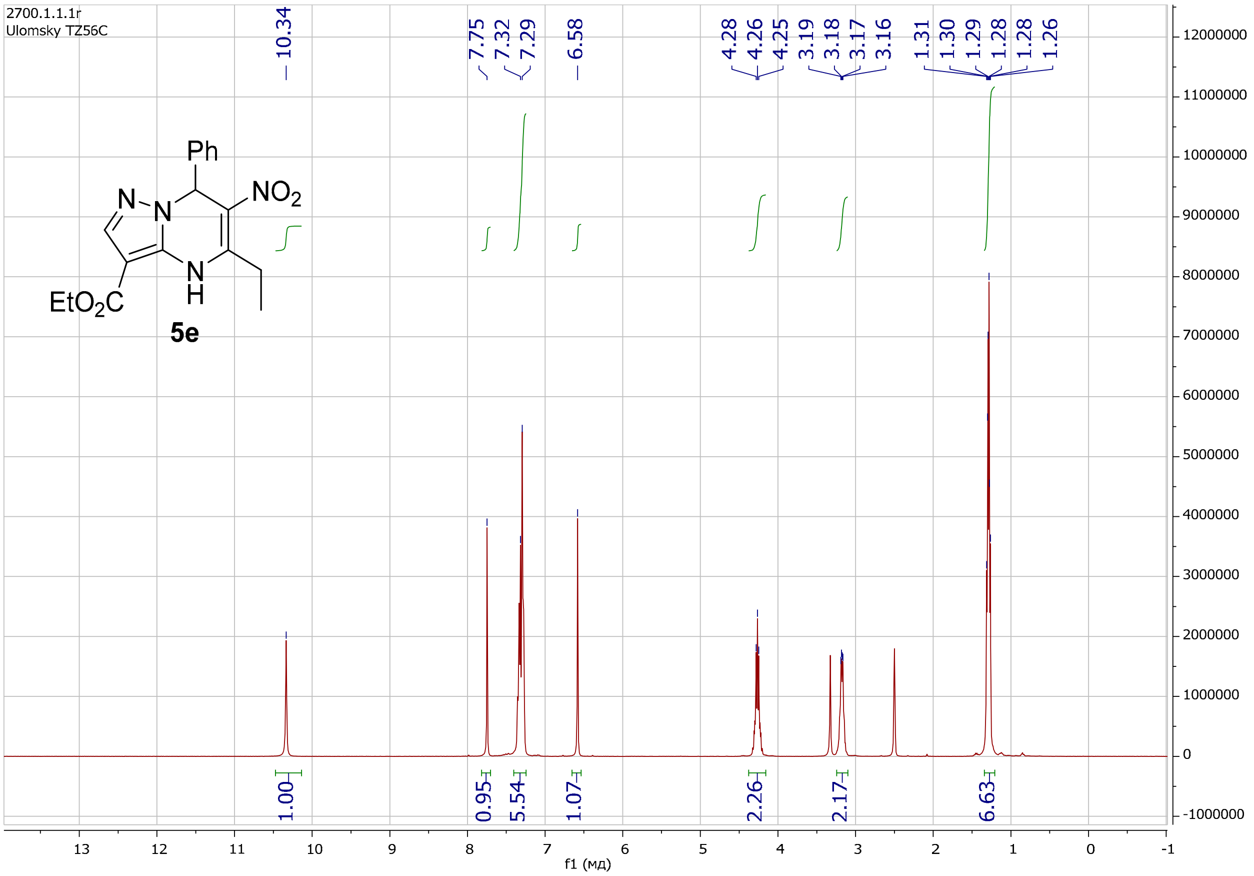
2700.1.1.1r
Ulomsky TZ56C
10.34
7.75
7.32
7.29
6.58
4.28
4.26
4.25
3.19
3.18
3.17
3.16
1.31
1.30
1.29
1.28
1.28
1.26
Ph
NO2
N
N
N
H
EtO2C
5e
1.00
0.95
5.54
1.07
2.26
2.17
6.63
f1 (мд)

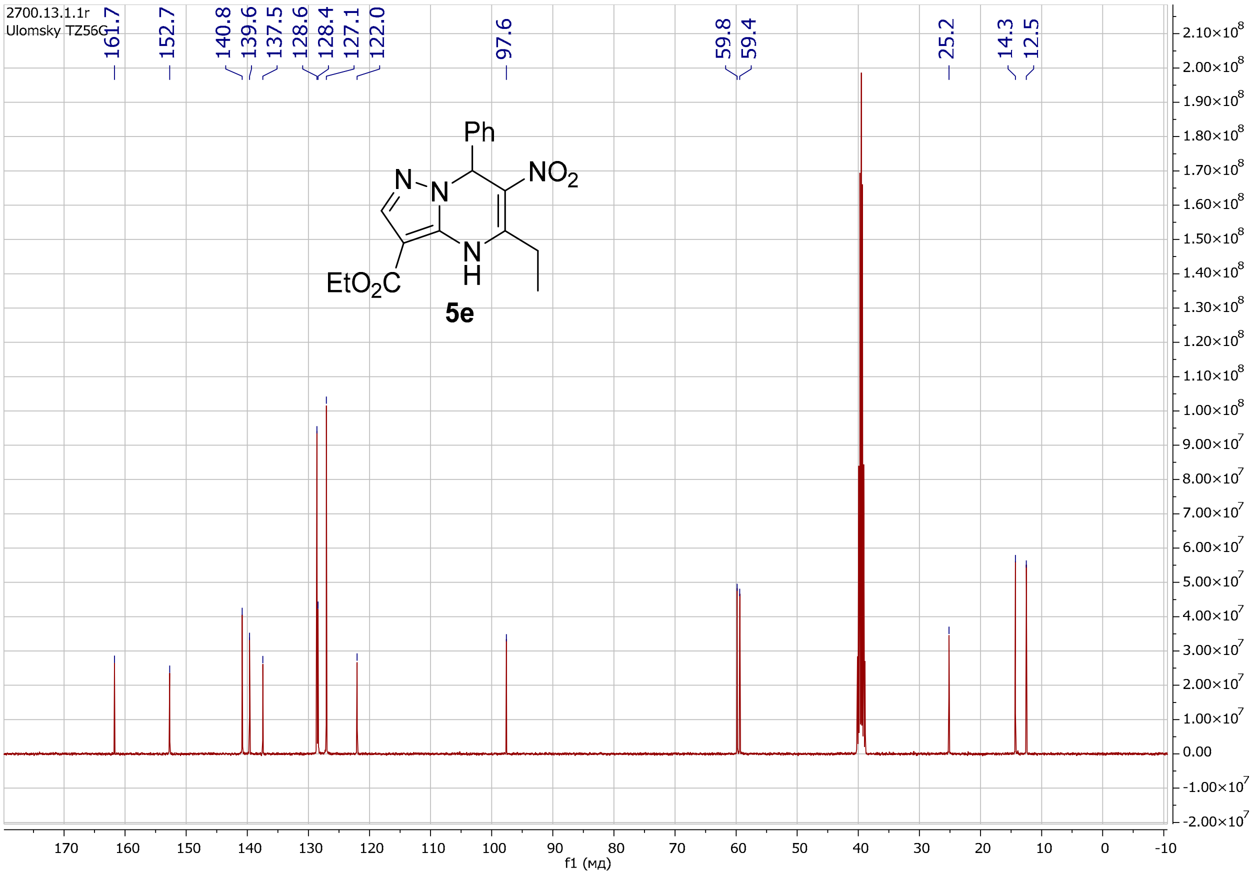
2700.13.1.1r
Ulomsky TZ56G
161.7
152.7
140.8
139.6
137.5
128.6
128.4
127.1
122.0
97.6
59.8
59.4
25.2
14.3
12.5
Ph
NO2
N
N
N
H
EtO2C
5e
f1 (мд)

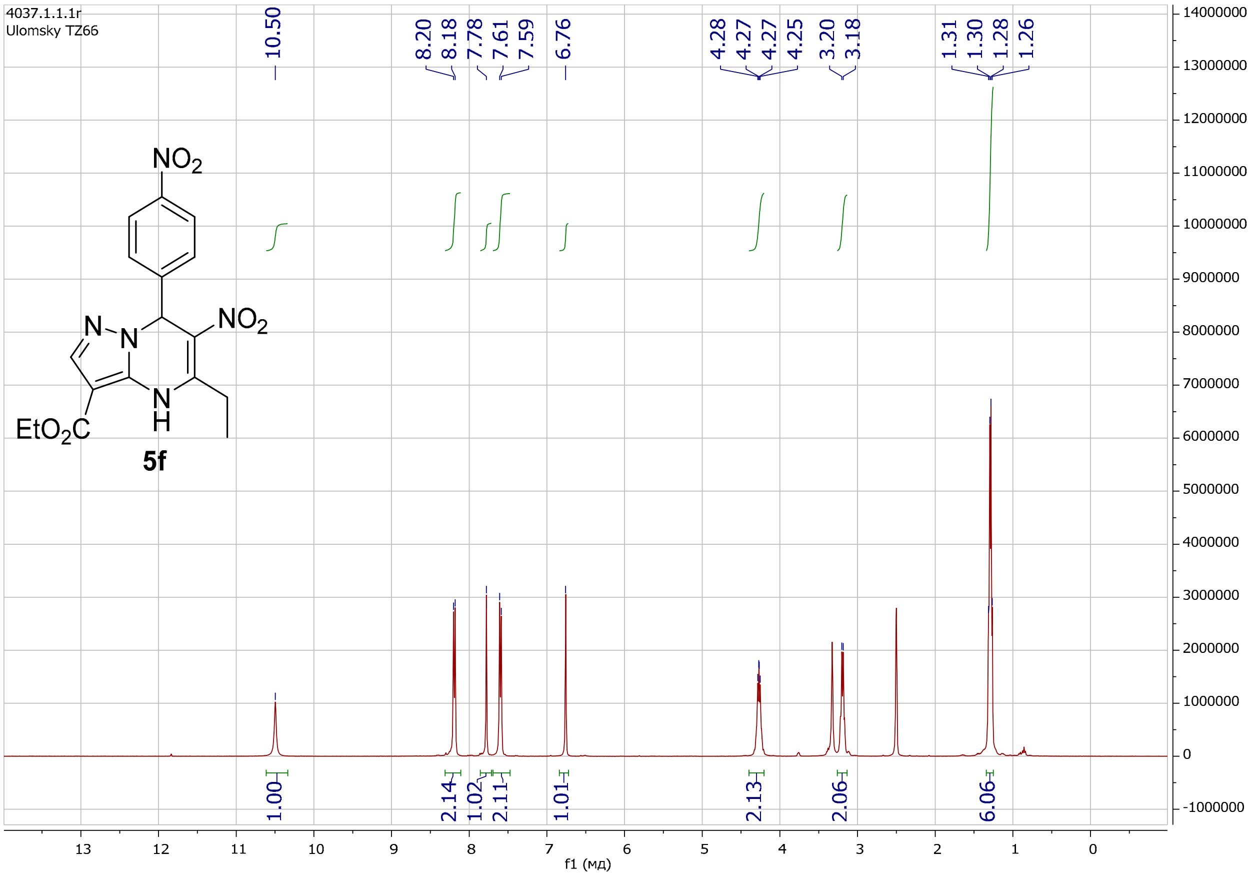
4037.1.1.1r
Ulomsky TZ66
10.50
8.20 8.18 7.78 7.61 7.59
6.76
4.28 4.27 4.27 4.25 3.20 3.18
1.31 1.30 1.28 1.26
1.00
2.14 1.02 2.11
1.01
2.13
2.06
6.06
NO2
NO2
N
N
N
H
EtO2C
5f
f1 (мд)

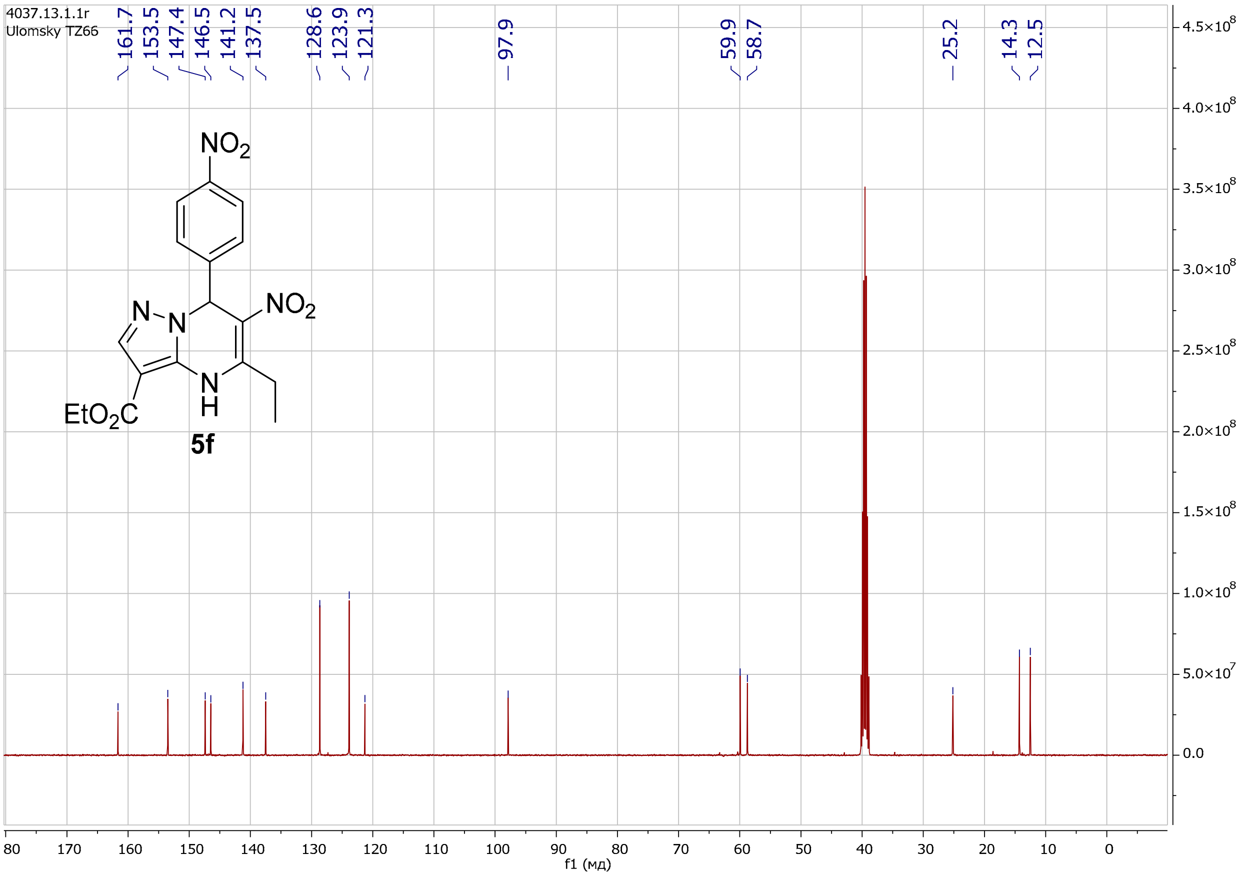
4037.13.1.1r
Ulomsky TZ66
161.7 153.5 147.4 146.5 141.2 137.5
128.6 123.9 121.3
97.9
59.9 58.7
25.2
14.3 12.5
NO2
NO2
N
N
N
H
EtO2C
5f
f1 (мд)

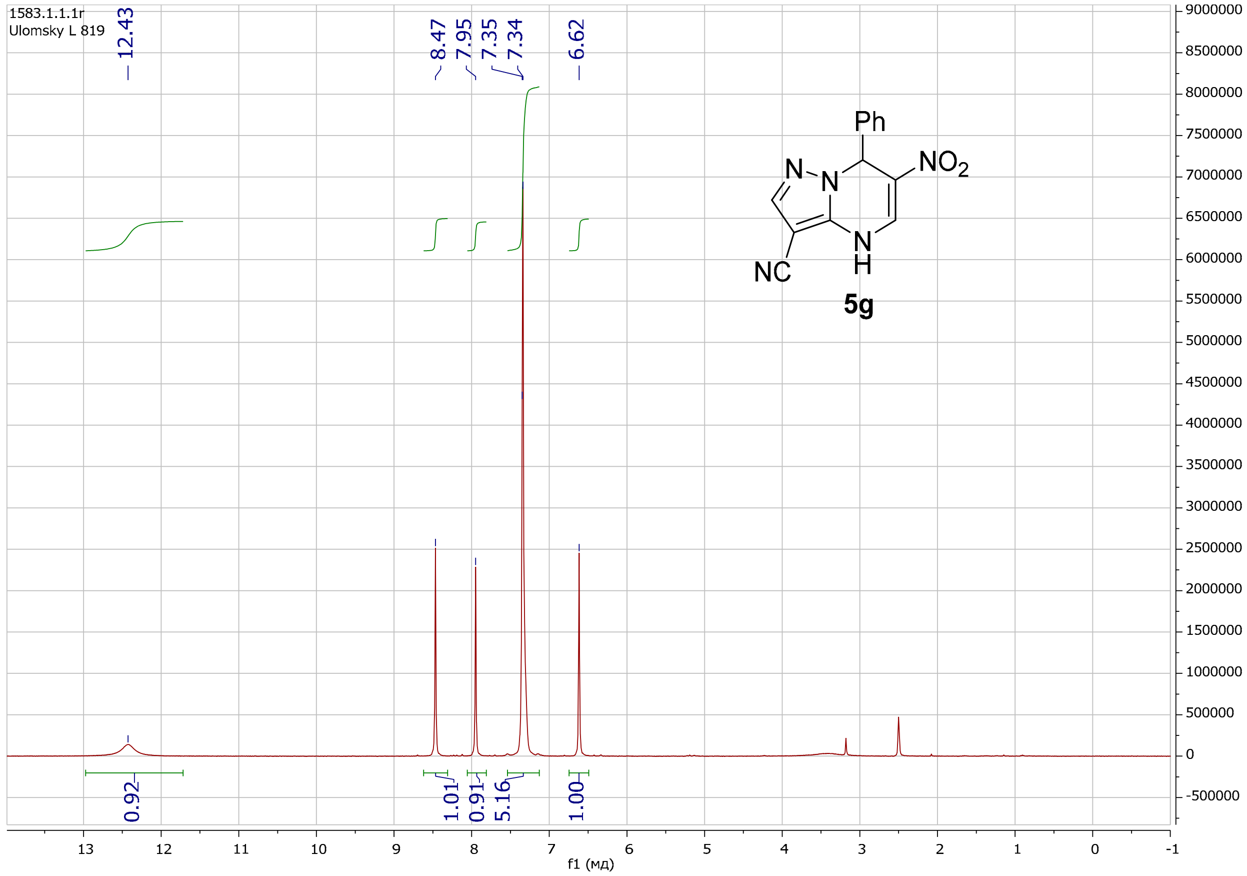
1583.1.1.1r
Ulomsky L 819
12.43
8.47
7.95
7.35
7.34
6.62
0.92
1.01
0.91
5.16
1.00
Ph
NO2
N
N
N
H
NC
5g
f1 (мд)

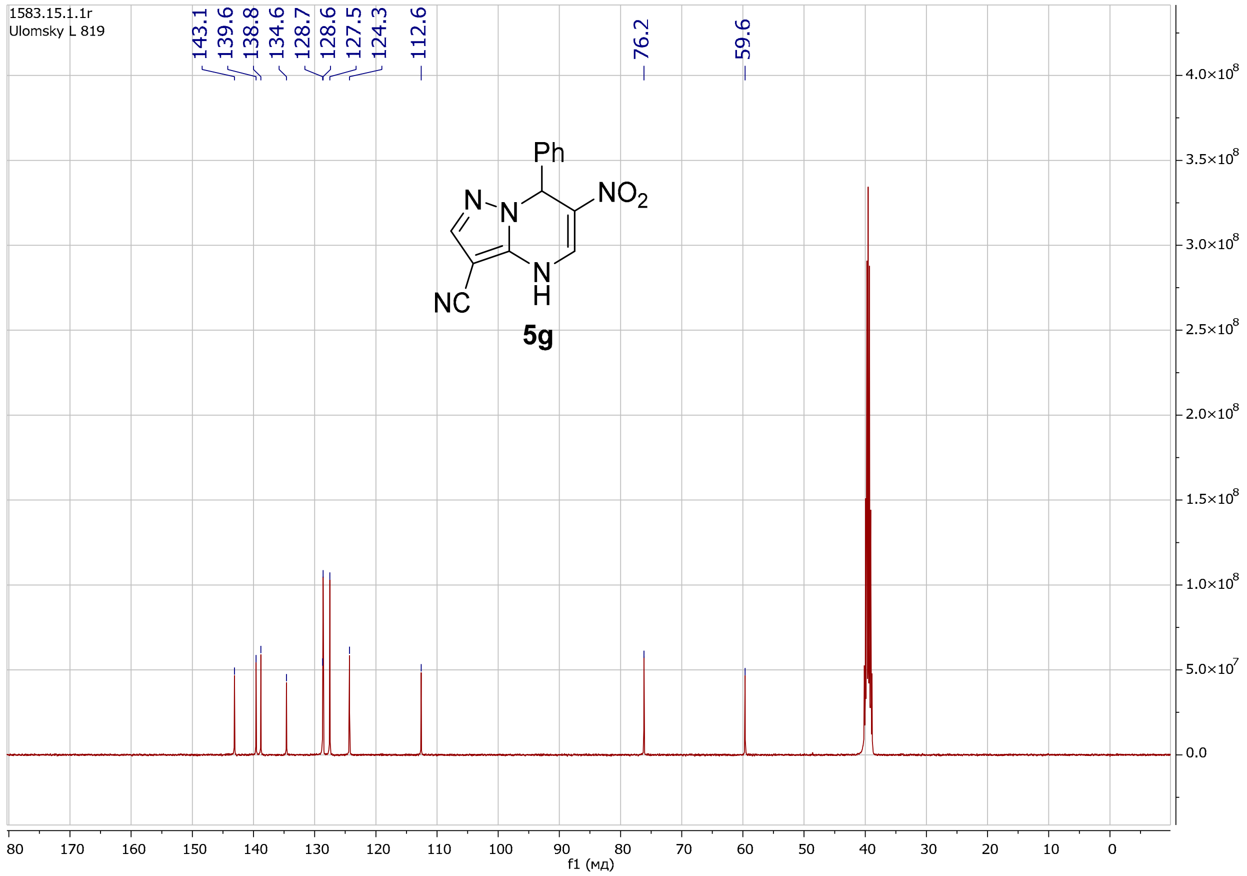
1583.15.1.1r
Ulomsky L 819
143.1
139.6
138.8
134.6
128.7
128.6
127.5
124.3
112.6
76.2
59.6
Ph
NO2
N
N
N
H
NC
5g
f1 (мд)

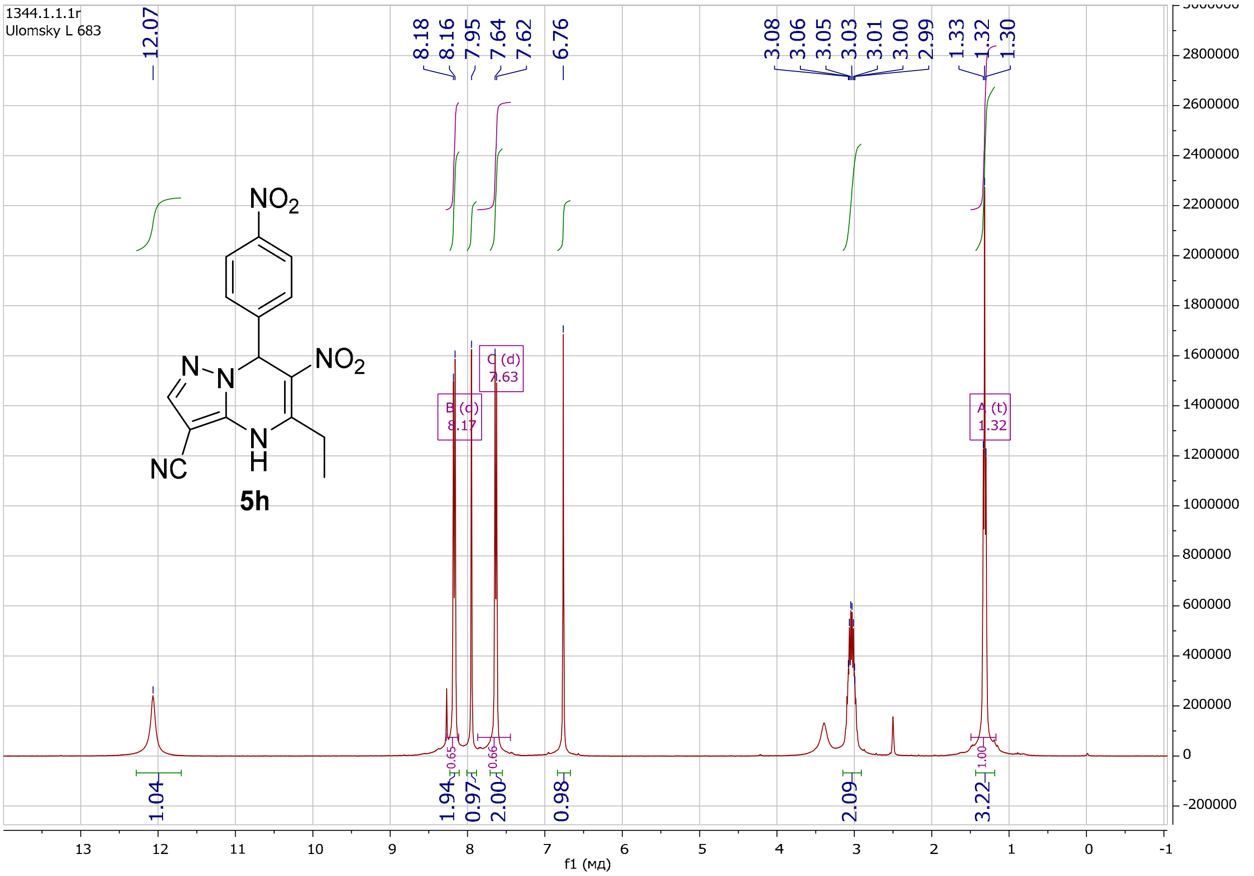
1344.1.1.1r
Ulomsky L 683
12.07
8.18
8.16
7.95
7.64
7.62
6.76
3.08
3.06
3.05
3.03
3.01
3.00
2.99
1.33
1.32
1.30
NO2
NO2
N-N
N
H
NC
5h
B (d)
8.17
C (d)
7.63
A (t)
1.32
1.04
1.94
0.97
2.00
0.98
2.09
3.22
f1 (мд)

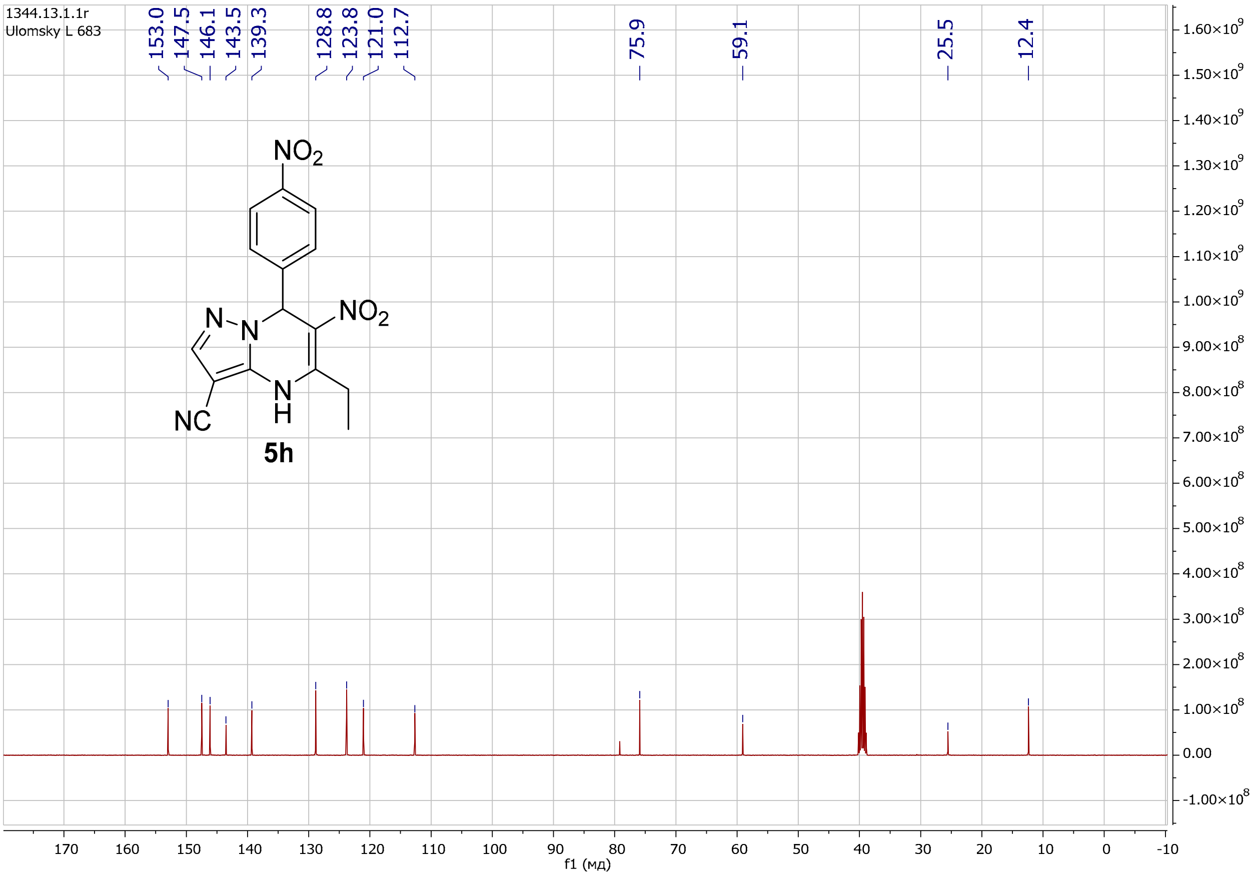
1344.13.1.1r
Ulomsky L 683
153.0
147.5
146.1
143.5
139.3
128.8
123.8
121.0
112.7
75.9
59.1
25.5
12.4
NO2
NO2
N-N
N
H
NC
5h
f1 (мд)

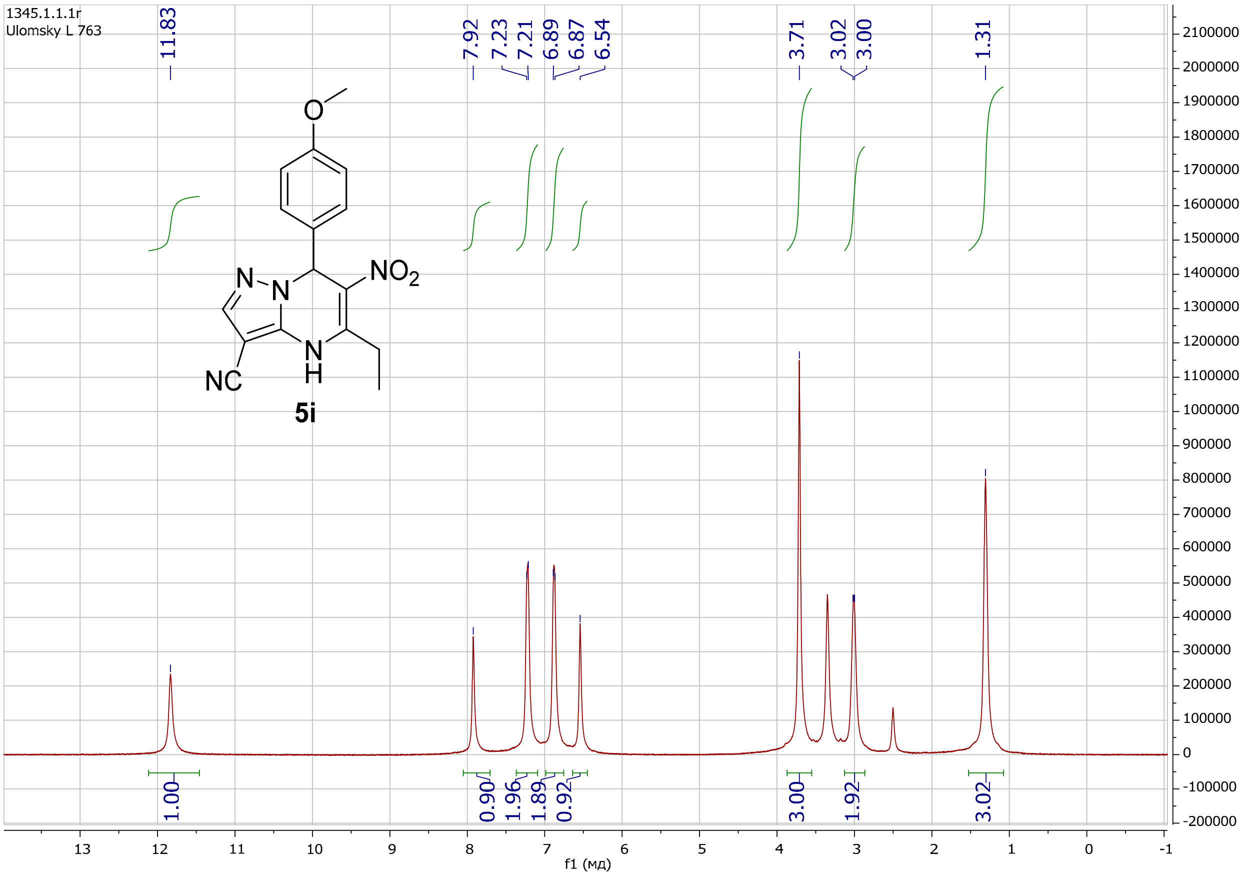
1345.1.1.1r
Ulomsky L 763
11.83
7.92
7.23
7.21
6.89
6.87
6.54
3.71
3.02
3.00
1.31
1.00
0.90
1.96
1.89
0.92
3.00
1.92
3.02
NO2
N
N
N
H
NC
O
5i
f1 (мд)

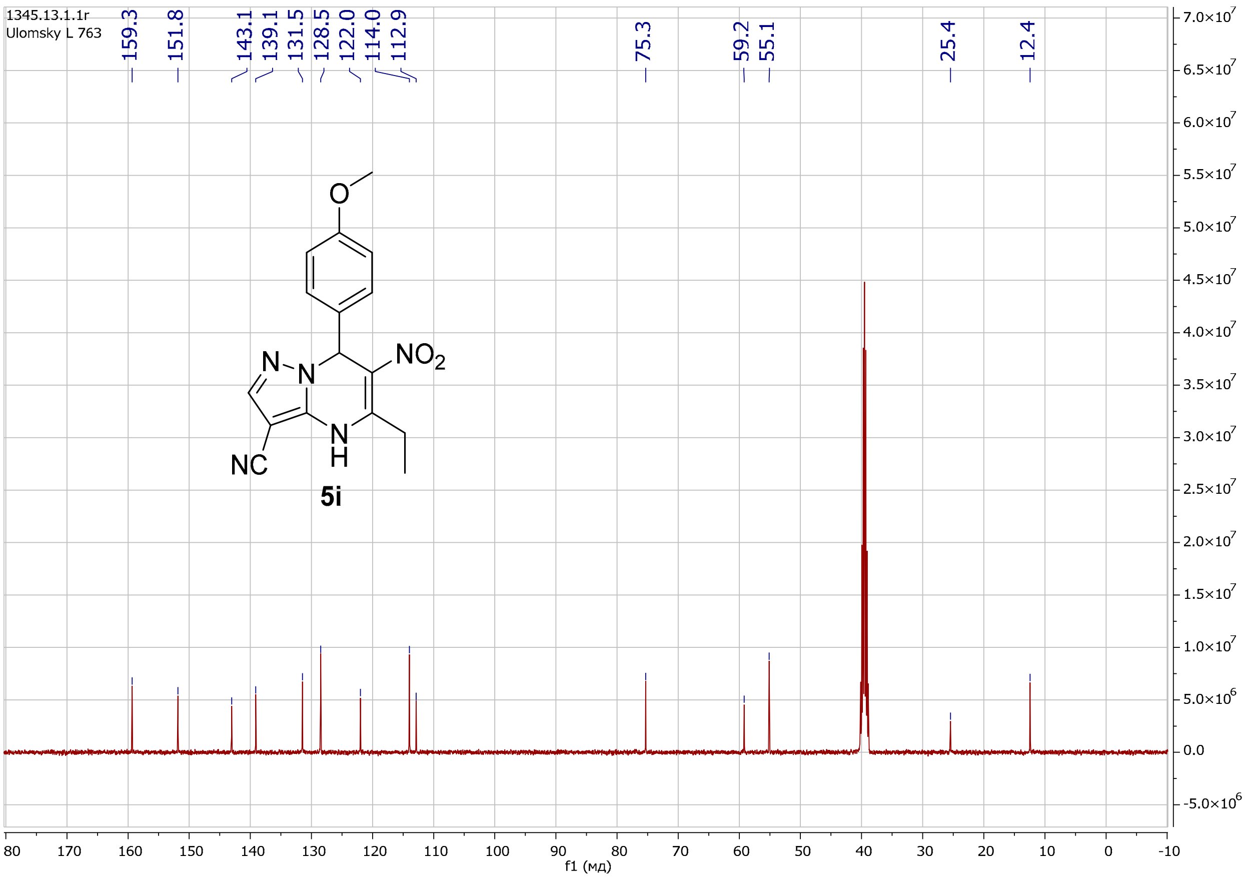
1345.13.1.1r
Ulomsky L 763
159.3
151.8
143.1
139.1
131.5
128.5
122.0
114.0
112.9
75.3
59.2
55.1
25.4
12.4
NO2
N
N
N
H
NC
O
5i
f1 (мд)

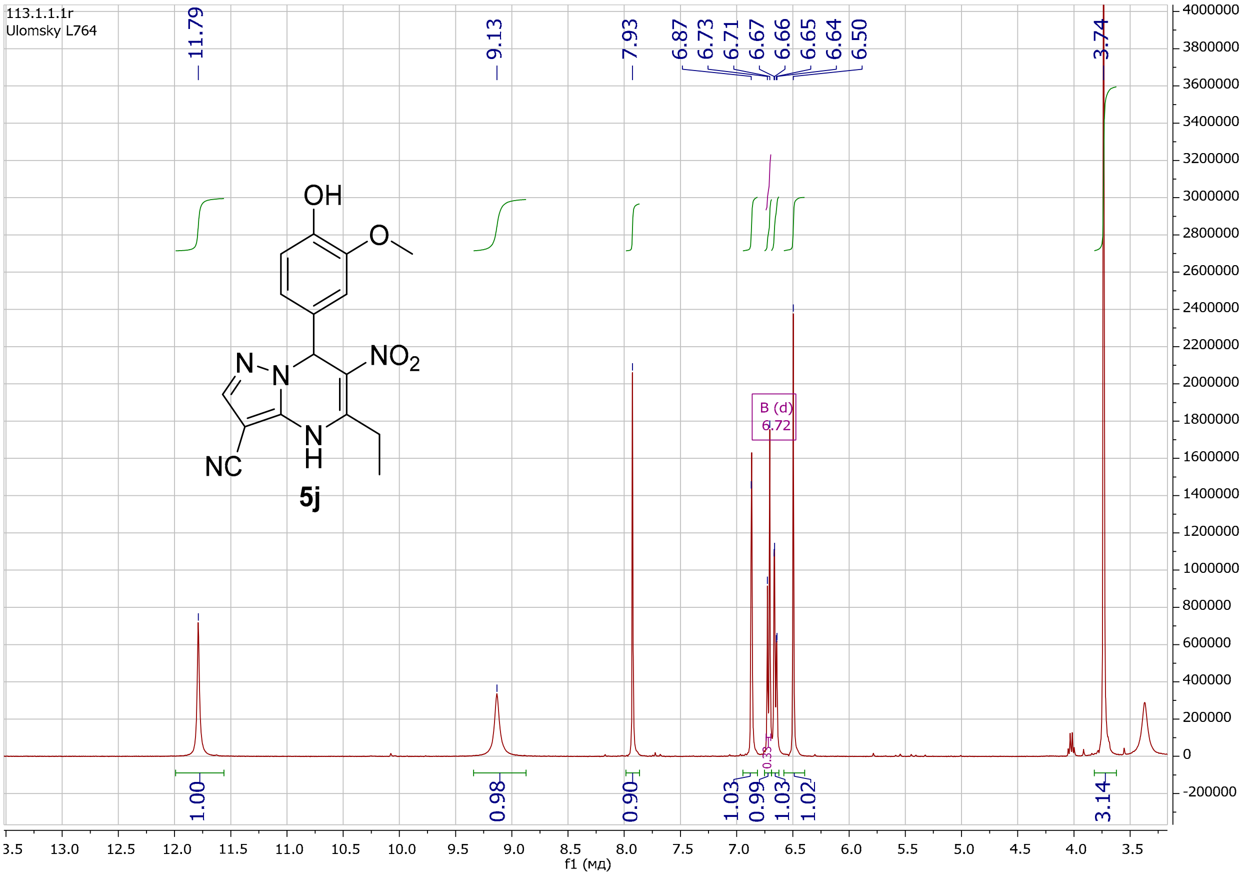
113.1.1.1r
Ulomsky L764
11.79
9.13
7.93
6.87
6.73
6.71
6.67
6.66
6.65
6.64
6.50
3.74
OH
O
NO2
N
N
N
H
NC
5j
B (d)
6.72
1.00
0.98
0.90
1.03
0.99
1.03
1.02
3.14
f1 (мд)

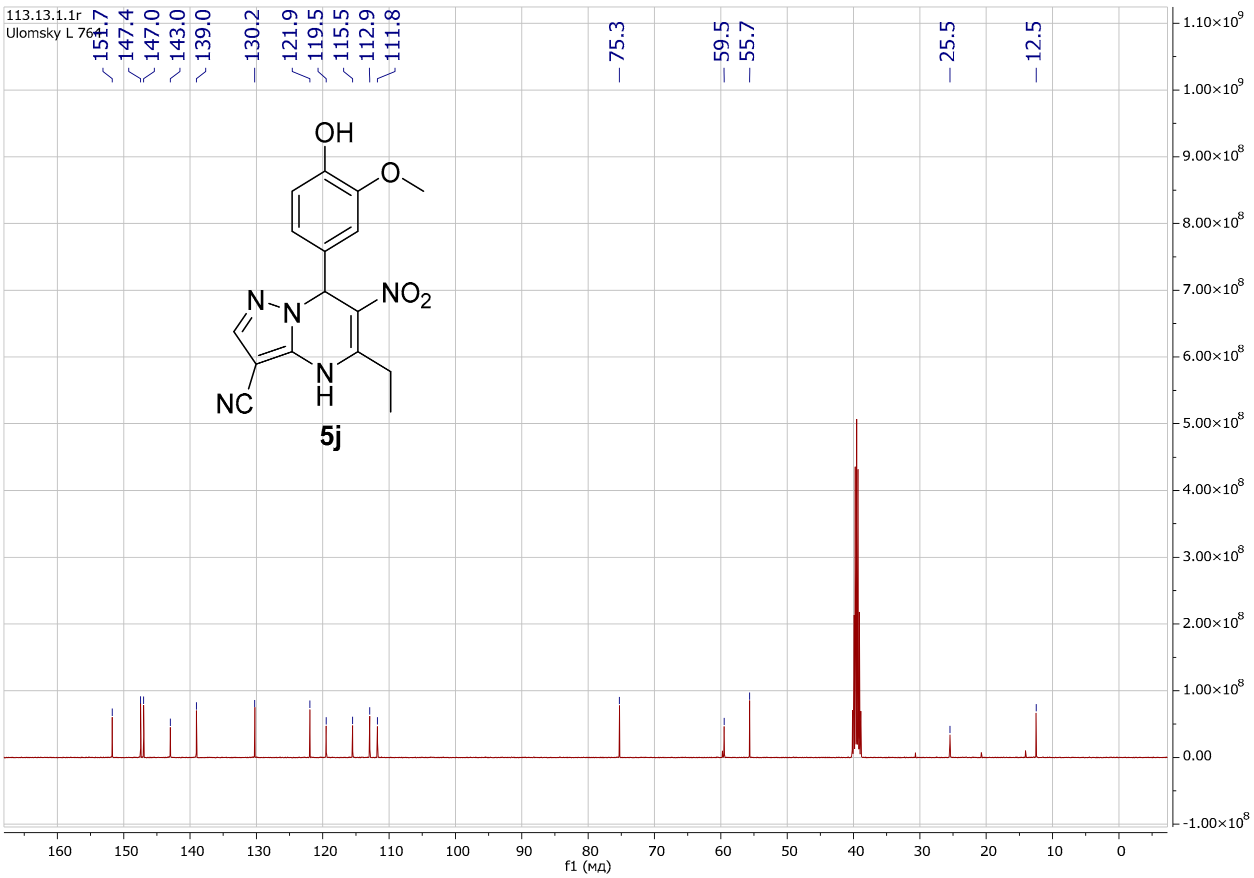
113.13.1.1r
Ulomsky L 764
151.7
147.4
147.0
143.0
139.0
130.2
121.9
119.5
115.5
112.9
111.8
75.3
59.5
55.7
25.5
12.5
OH
O
NO2
N
N
N
H
NC
5j
f1 (мд)

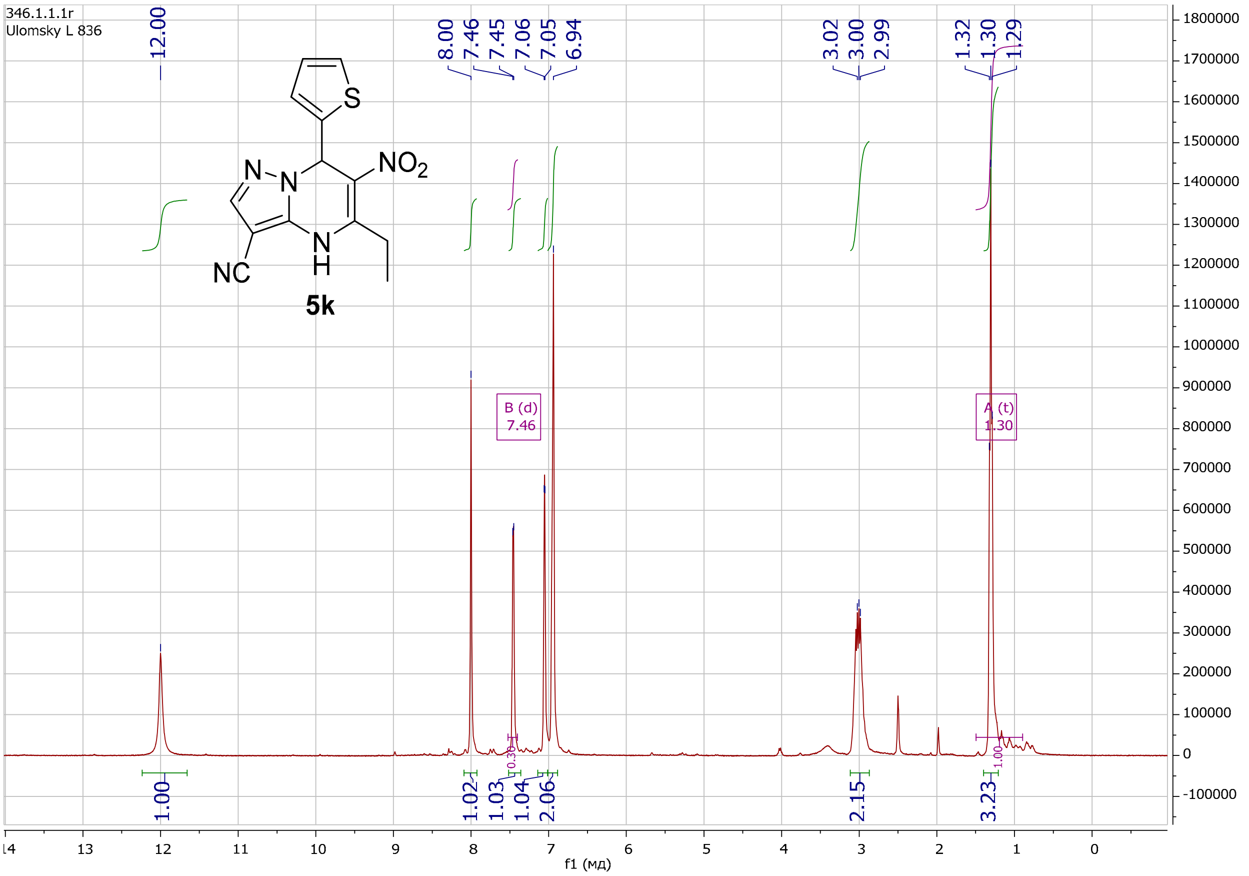
346.1.1.1r
Ulomsky L 836
12.00
8.00
7.46
7.45
7.06
7.05
6.94
3.02
3.00
2.99
1.32
1.30
1.29
NO2
N
N
N
H
NC
S
5k
B (d)
7.46
A (t)
1.30
1.00
1.02
1.03
1.04
2.06
2.15
3.23
f1 (мд)

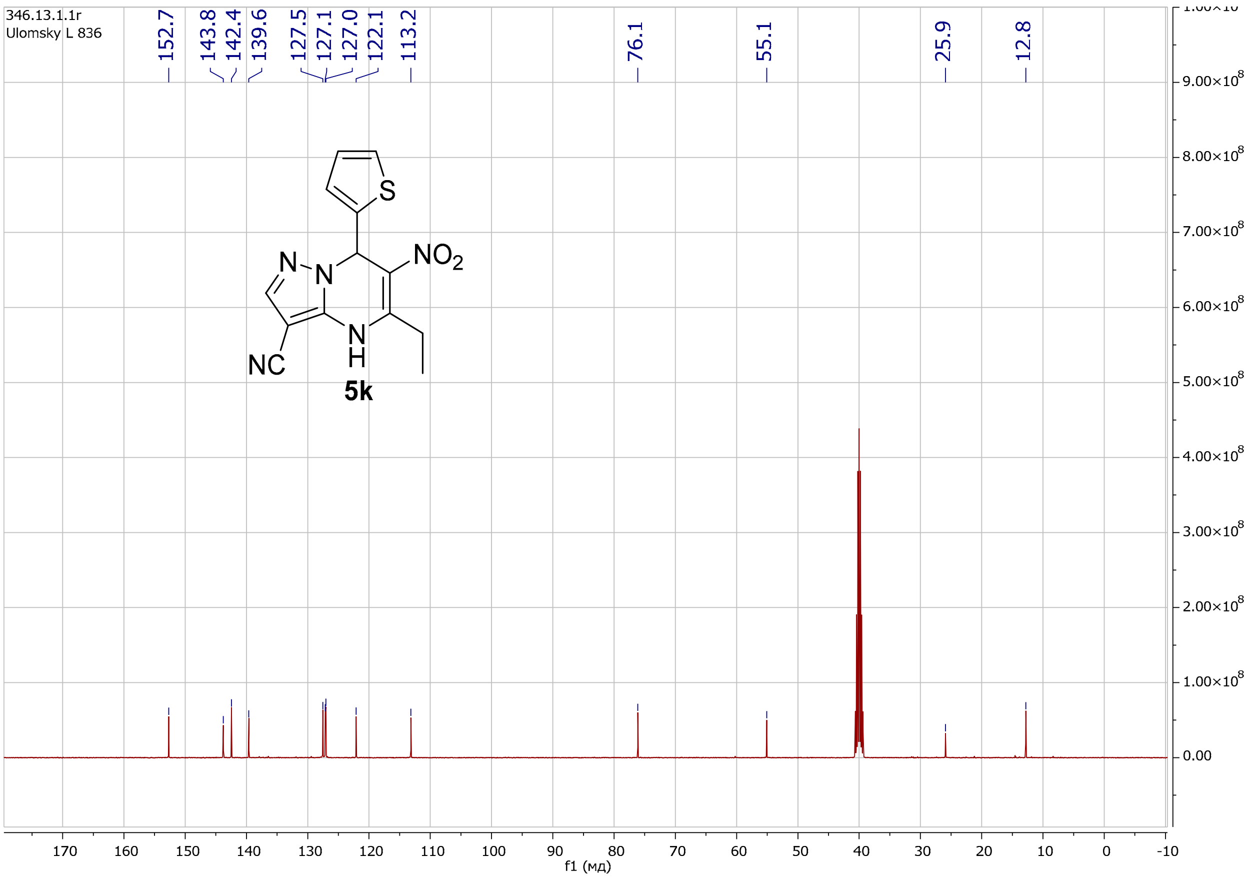
346.13.1.1r
Ulomsky L 836
152.7
143.8
142.4
139.6
127.5
127.1
127.0
122.1
113.2
76.1
55.1
25.9
12.8
NO2
NC
S
5k
f1 (мд)

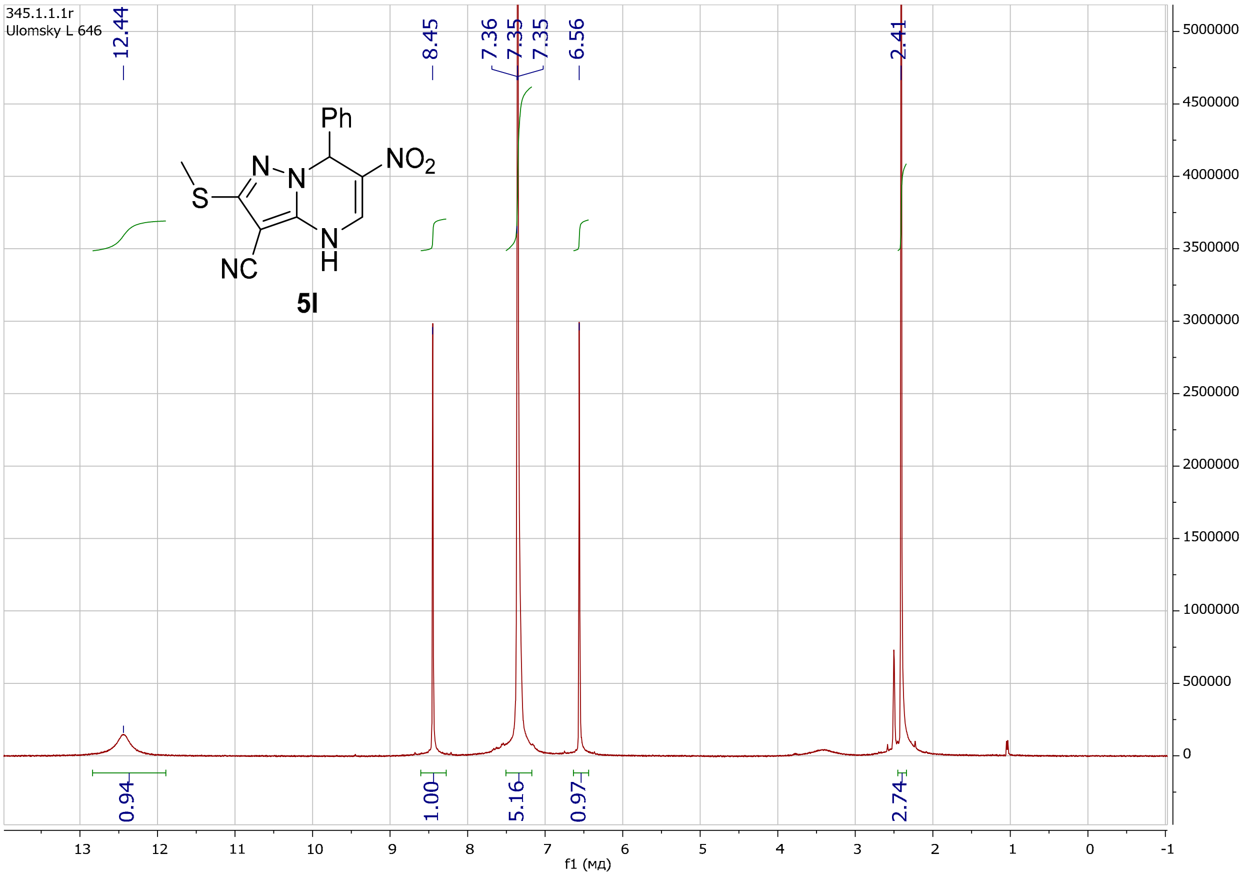
345.1.1.1r
Ulomsky L 646
12.44
8.45
7.36
7.35
7.35
6.56
2.41
Ph
NO2
N
N
S
NC
N
H
5l
0.94
1.00
5.16
0.97
2.74
f1 (мд)

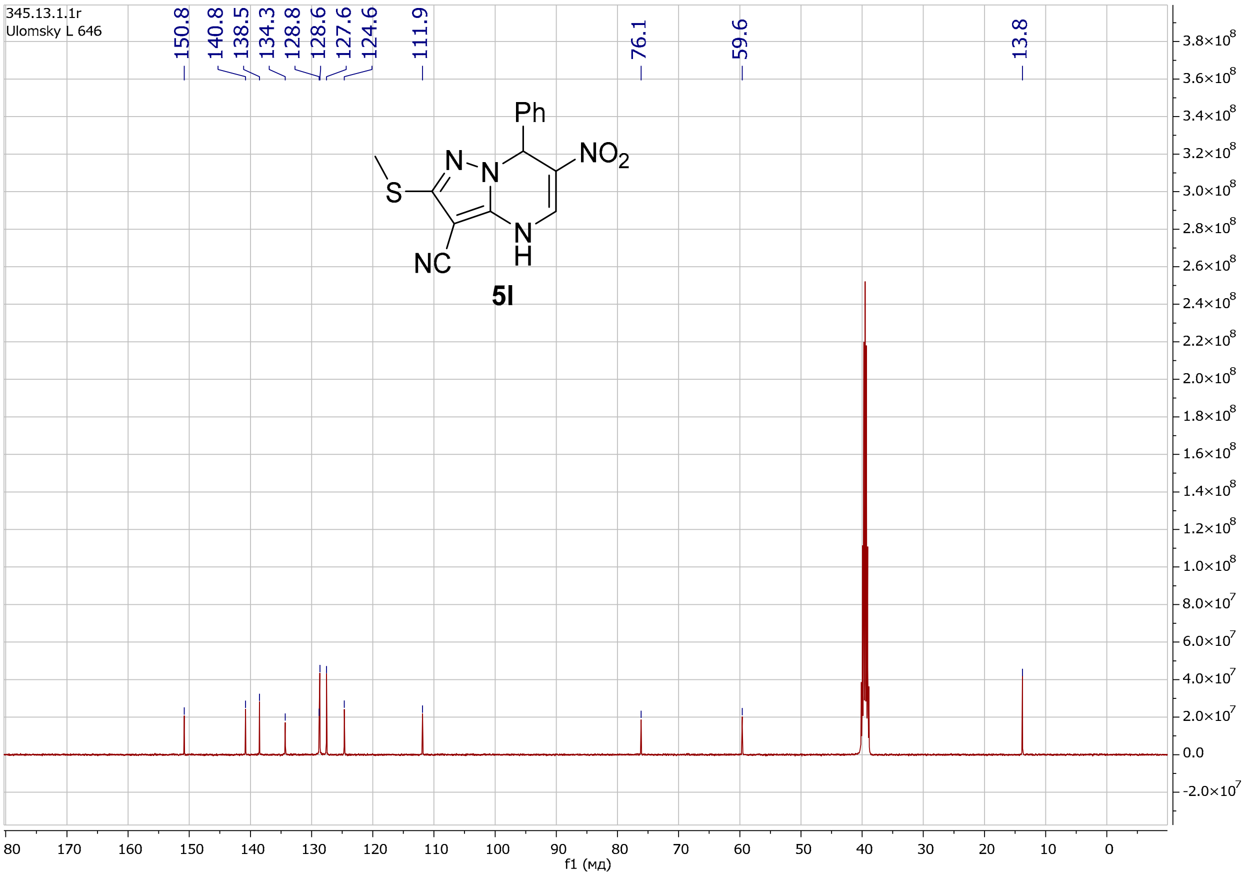
345.13.1.1r
Ulomsky L 646
150.8
140.8
138.5
134.3
128.8
128.6
127.6
124.6
111.9
76.1
59.6
13.8
Ph
NO2
N
N
S
NC
N
H
5l
f1 (мд)

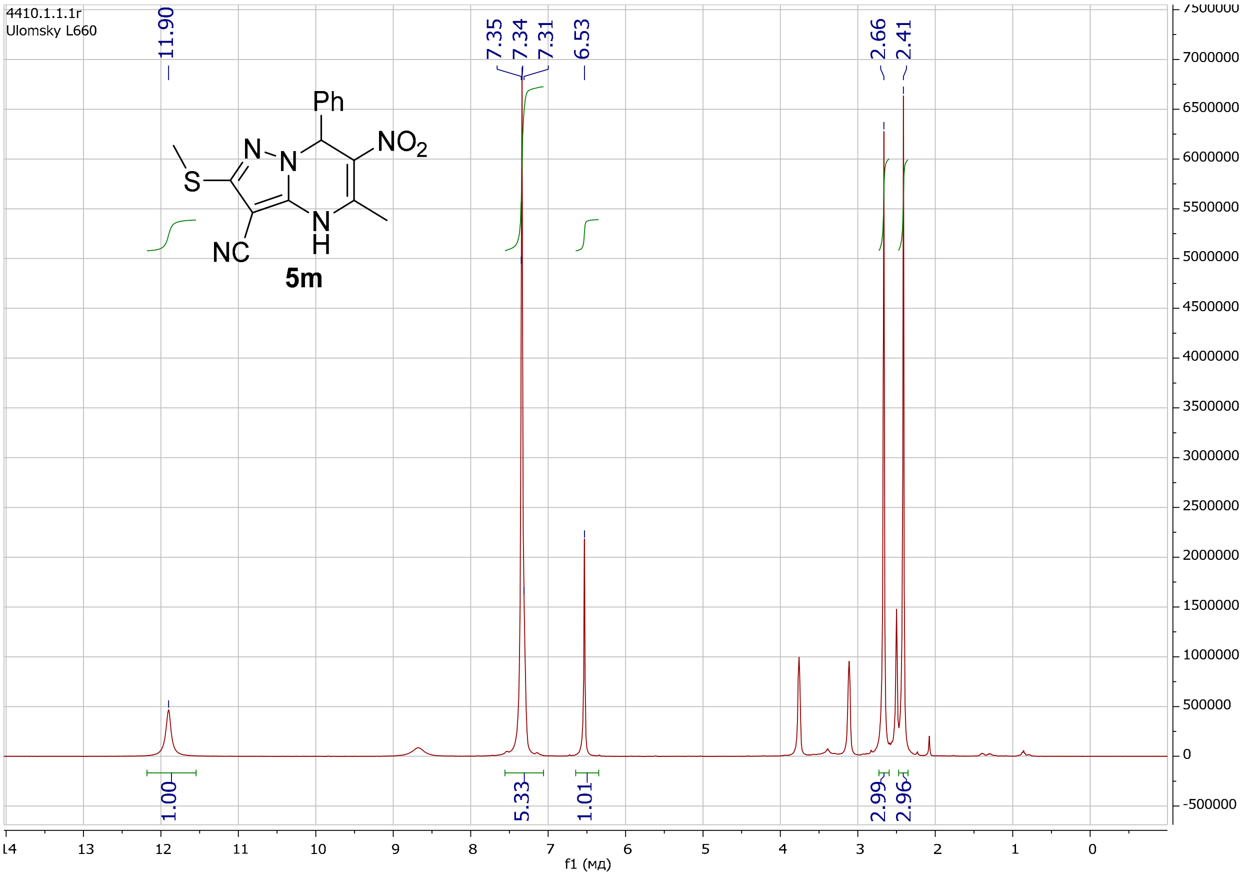
4410.1.1.1r
Ulomsky L660
11.90
7.35
7.34
7.31
6.53
2.66
2.41
Ph
NO2
N
N
S
NC
N
H
5m
1.00
5.33
1.01
2.99
2.96
f1 (мд)

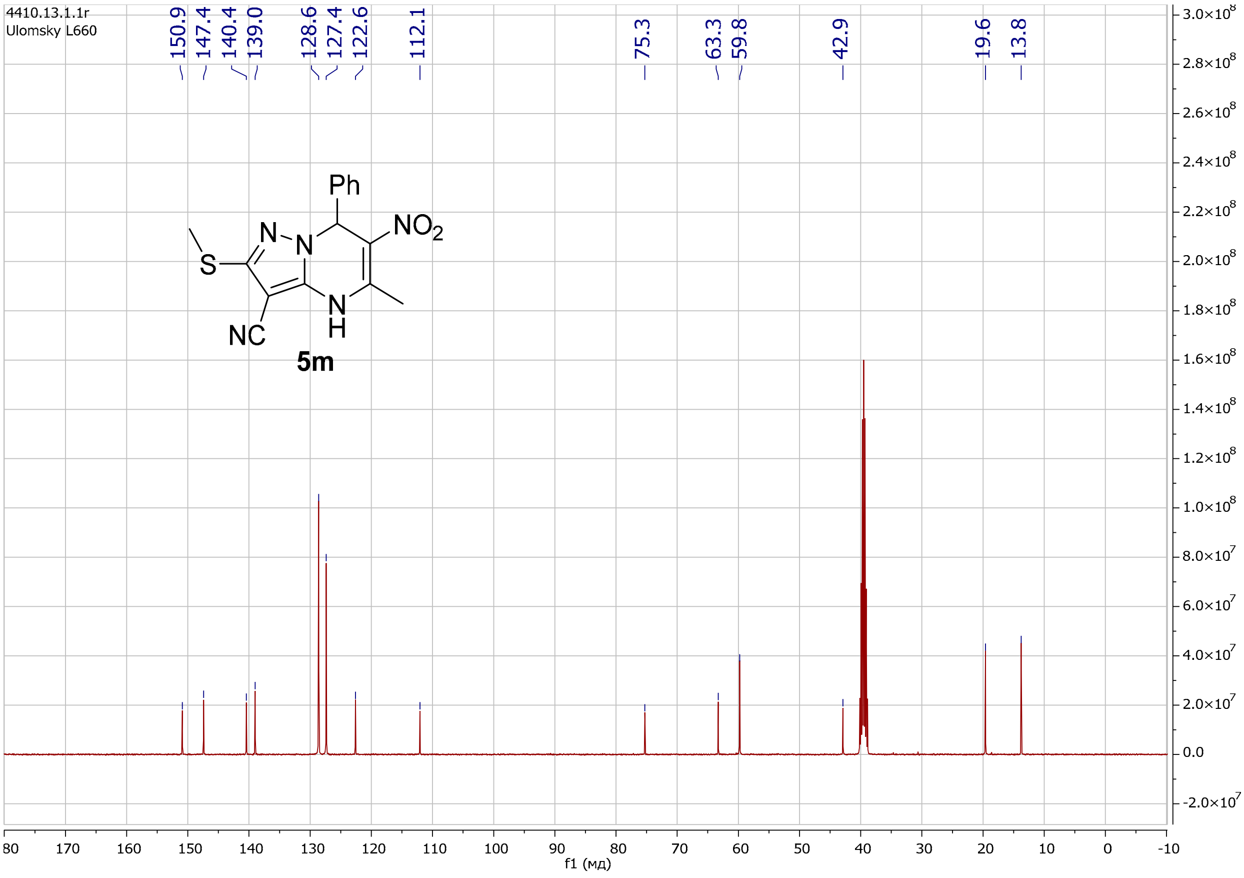
4410.13.1.1r
Ulomsky L660
150.9
147.4
140.4
139.0
128.6
127.4
122.6
112.1
75.3
63.3
59.8
42.9
19.6
13.8
Ph
NO2
N
N
S
NC
N
H
5m
f1 (мд)

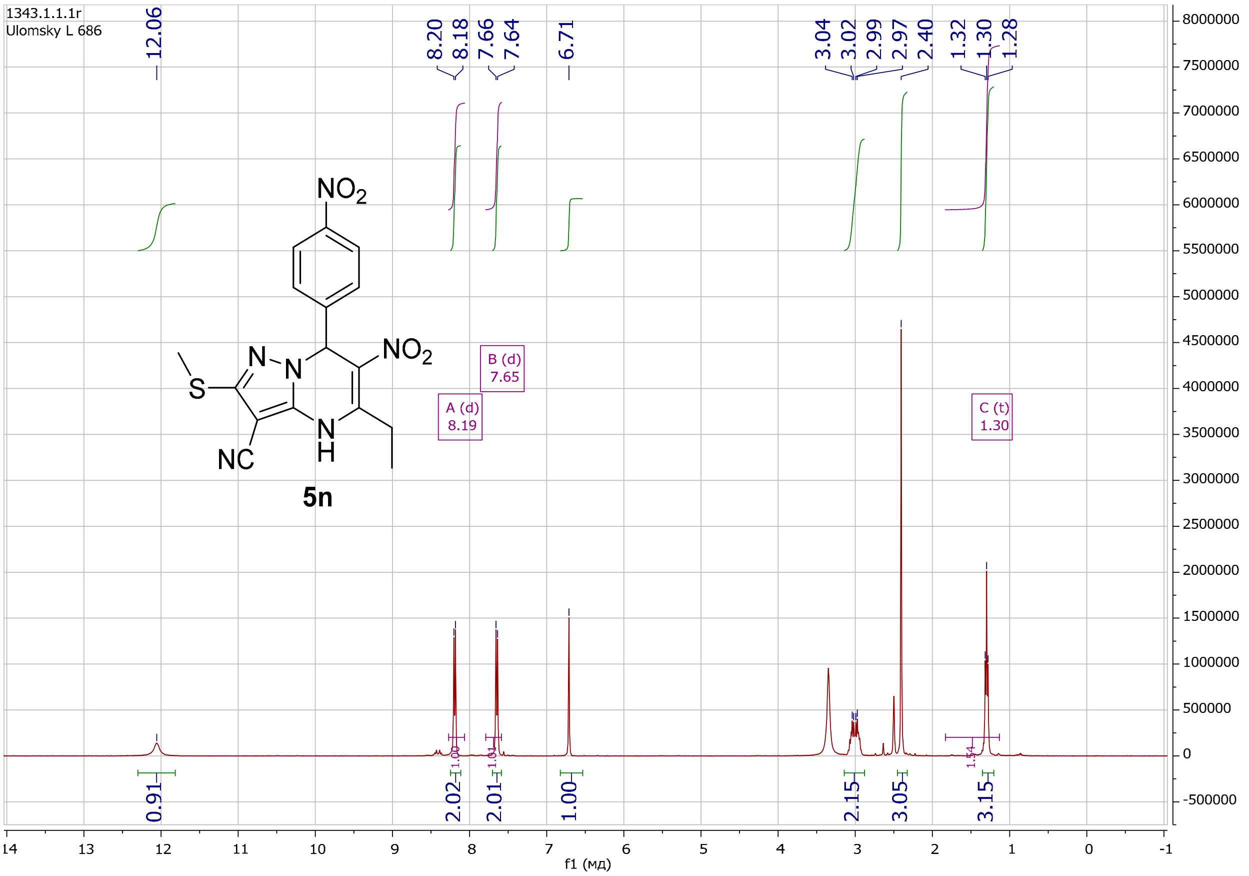
1343.1.1.1r
Ulomsky L 686
12.06
8.20
8.18
7.66
7.64
6.71
3.04
3.02
2.99
2.97
2.40
1.32
1.30
1.28
NO2
NO2
N
N
S
NC
N
H
5n
A (d)
8.19
B (d)
7.65
C (t)
1.30
0.91
2.02
2.01
1.00
2.15
3.05
3.15
f1 (мд)

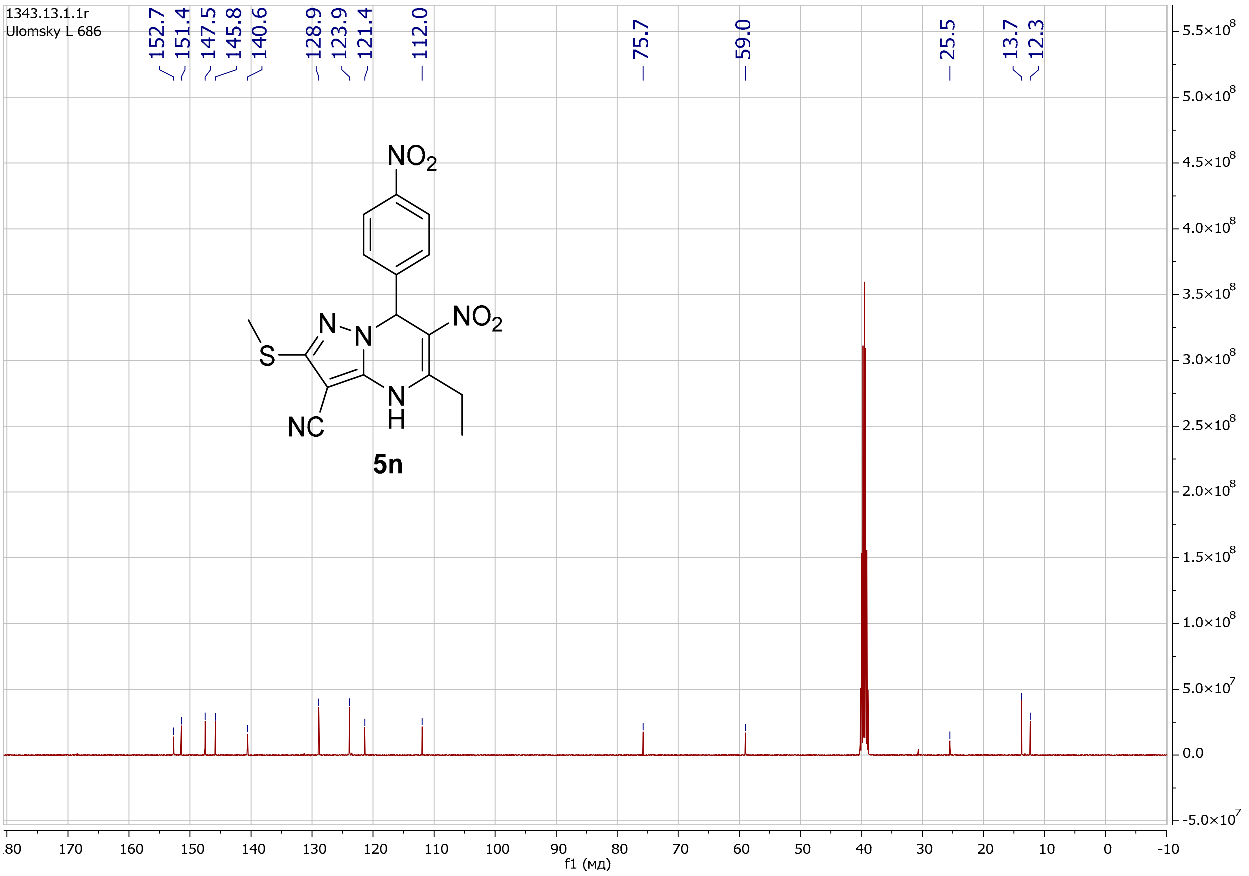
1343.13.1.1r
Ulomsky L 686
152.7
151.4
147.5
145.8
140.6
128.9
123.9
121.4
112.0
75.7
59.0
25.5
13.7
12.3
NO2
NO2
N
N
S
NC
N
H
5n
f1 (мд)

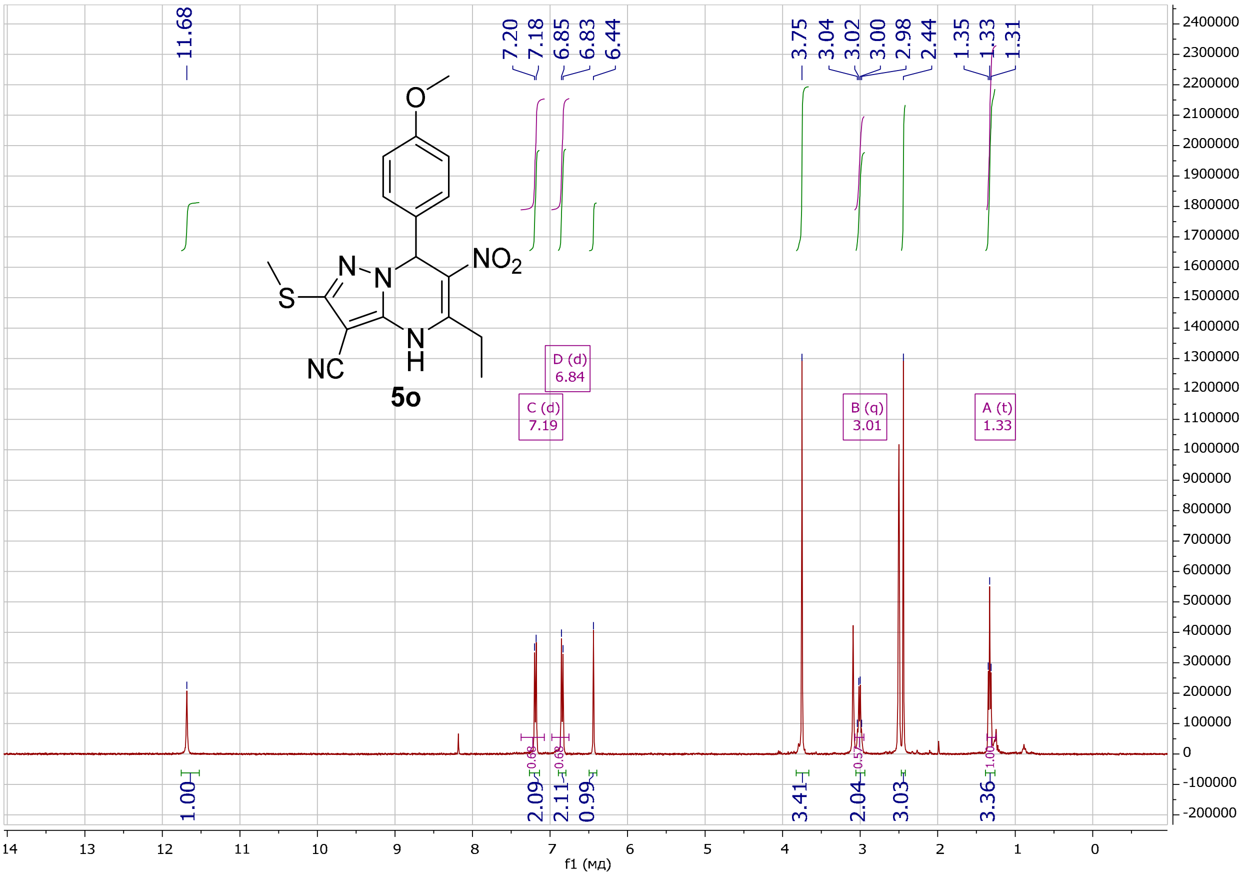
11.68
7.20
7.18
6.85
6.83
6.44
3.75
3.04
3.02
3.00
2.98
2.44
1.35
1.33
1.31
NO2
S
N
N
NC
N
H
O
5o
D (d)
6.84
C (d)
7.19
B (q)
3.01
A (t)
1.33
1.00
2.09
2.11
0.99
3.41
2.04
3.03
3.36
f1 (мд)

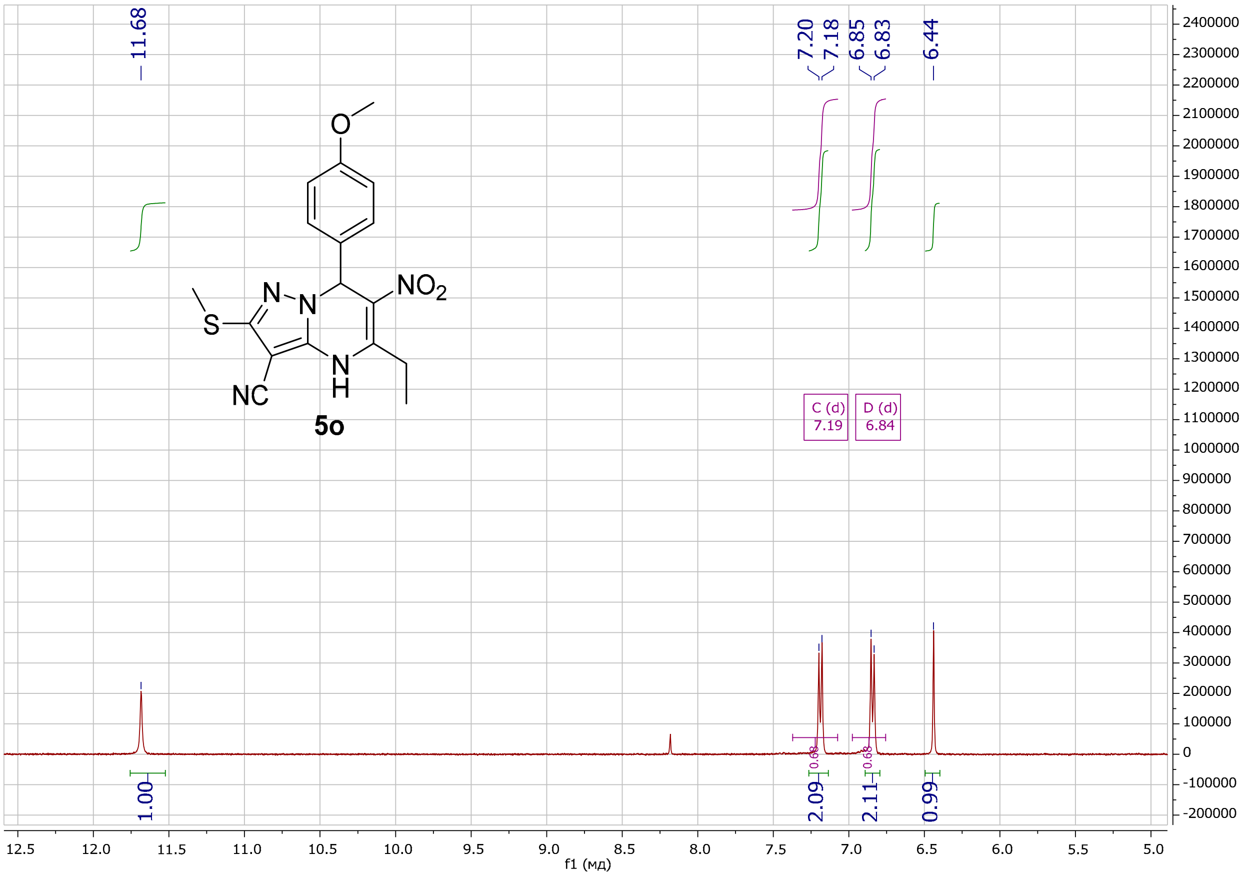
11.68
7.20
7.18
6.85
6.83
6.44
NO2
S
N
N
NC
N
H
O
5o
C (d)
7.19
D (d)
6.84
1.00
2.09
2.11
0.99
f1 (мд)

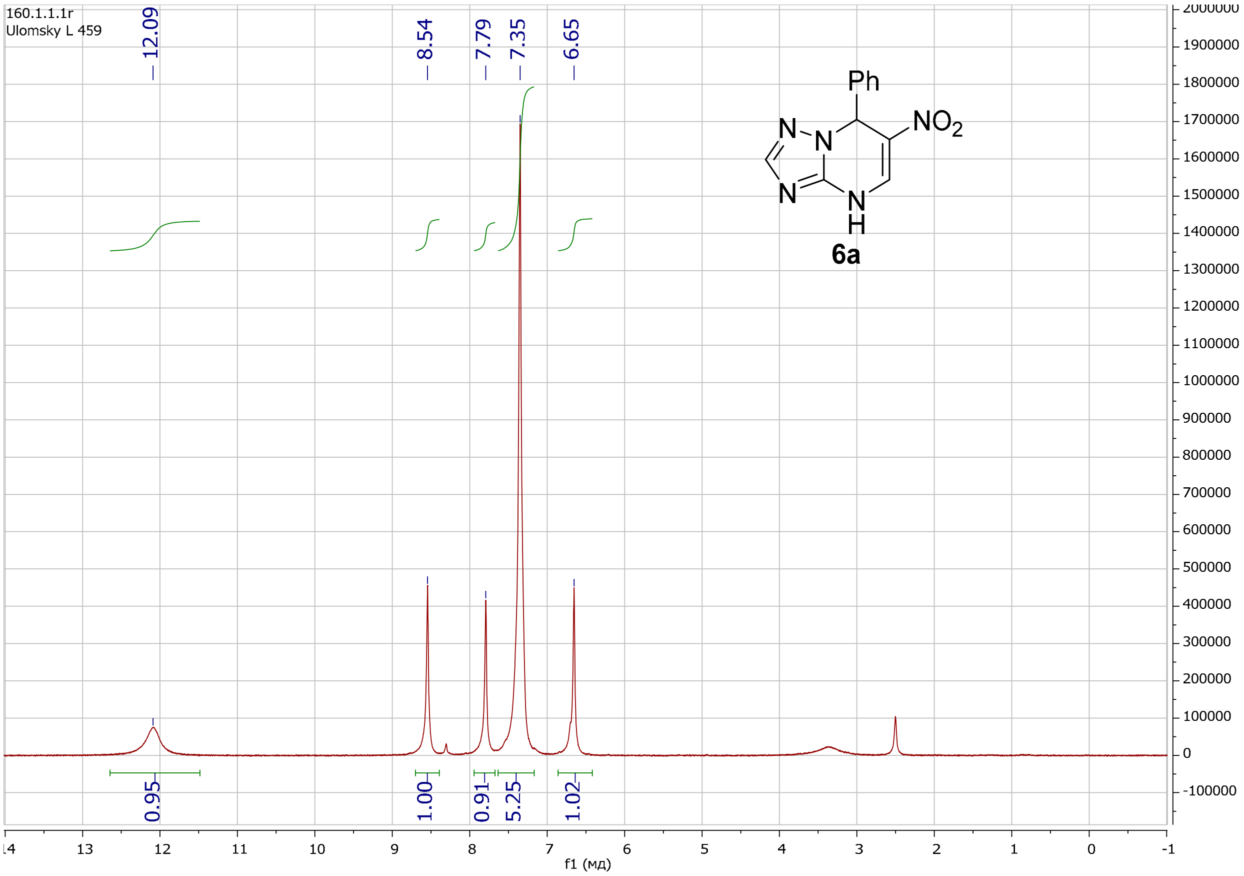
160.1.1.1r
Ulomsky L 459
12.09
8.54
7.79
7.35
6.65
Ph
NO2
N
N
N
N
H
6a
0.95
1.00
0.91
5.25
1.02
f1 (мд)

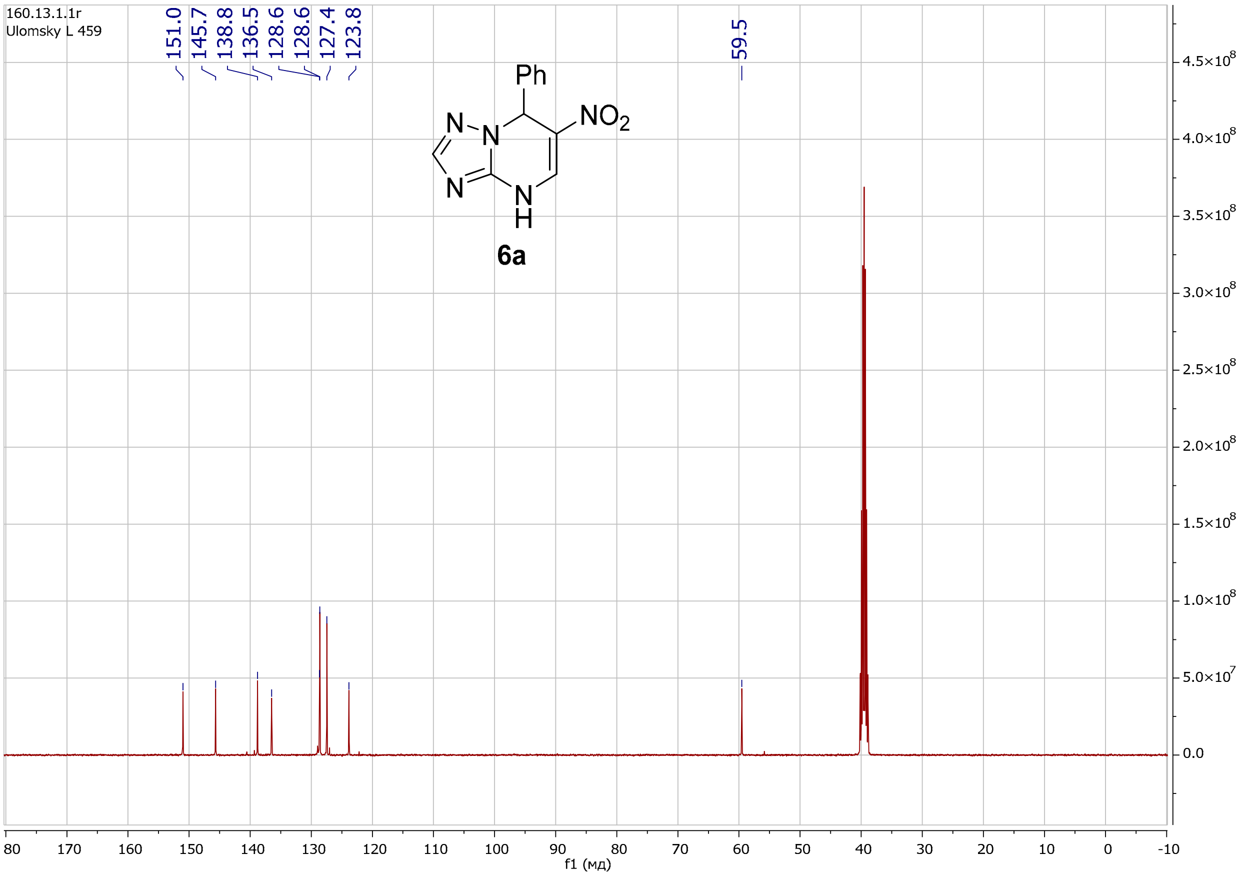
160.13.1.1r
Ulomsky L 459
151.0
145.7
138.8
136.5
128.6
128.6
127.4
123.8
59.5
Ph
NO2
N
N
N
N
H
6a
f1 (мд)

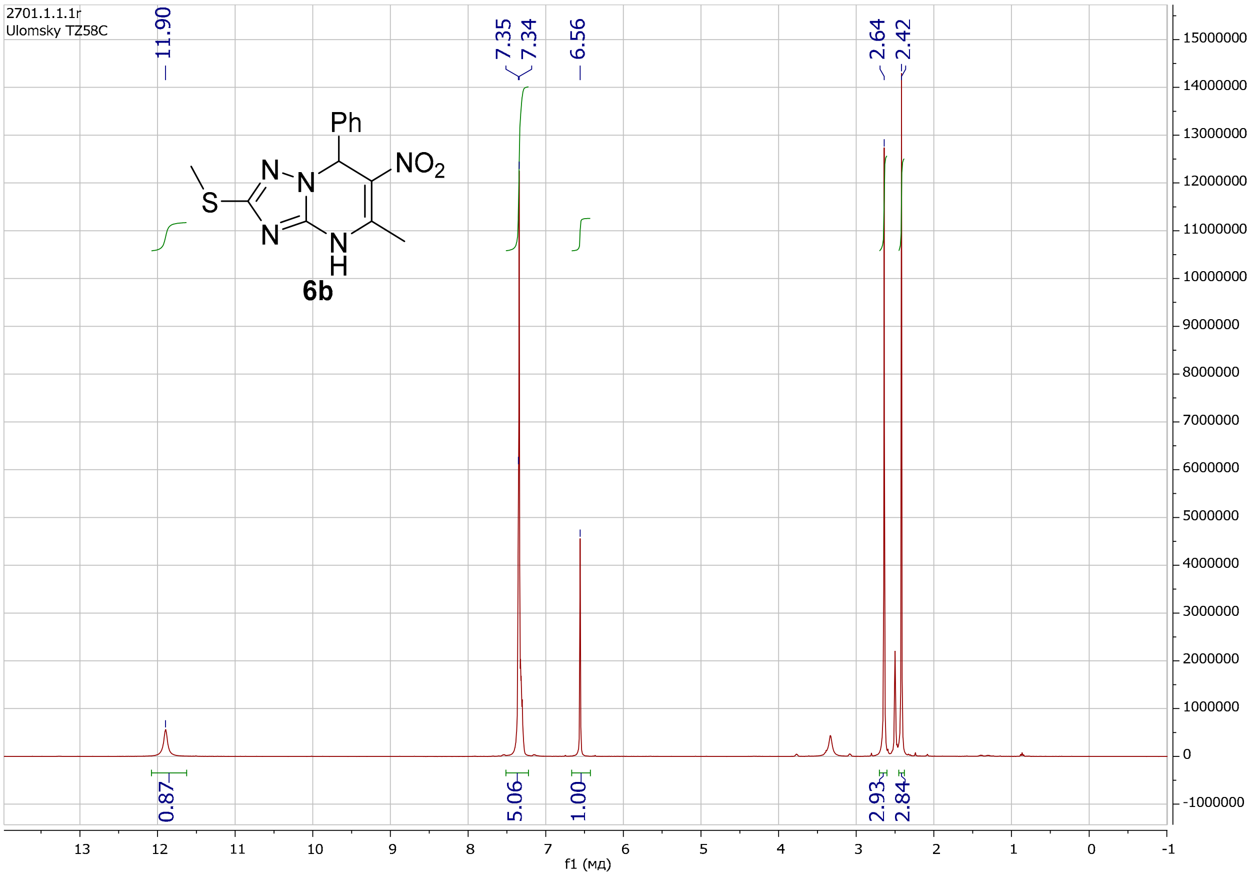
2701.1.1.1r
Ulomsky TZ58C
11.90
7.35
7.34
6.56
2.64
2.42
Ph
NO2
N
N
N
S
N
H
6b
0.87
5.06
1.00
2.93
2.84
f1 (мд)

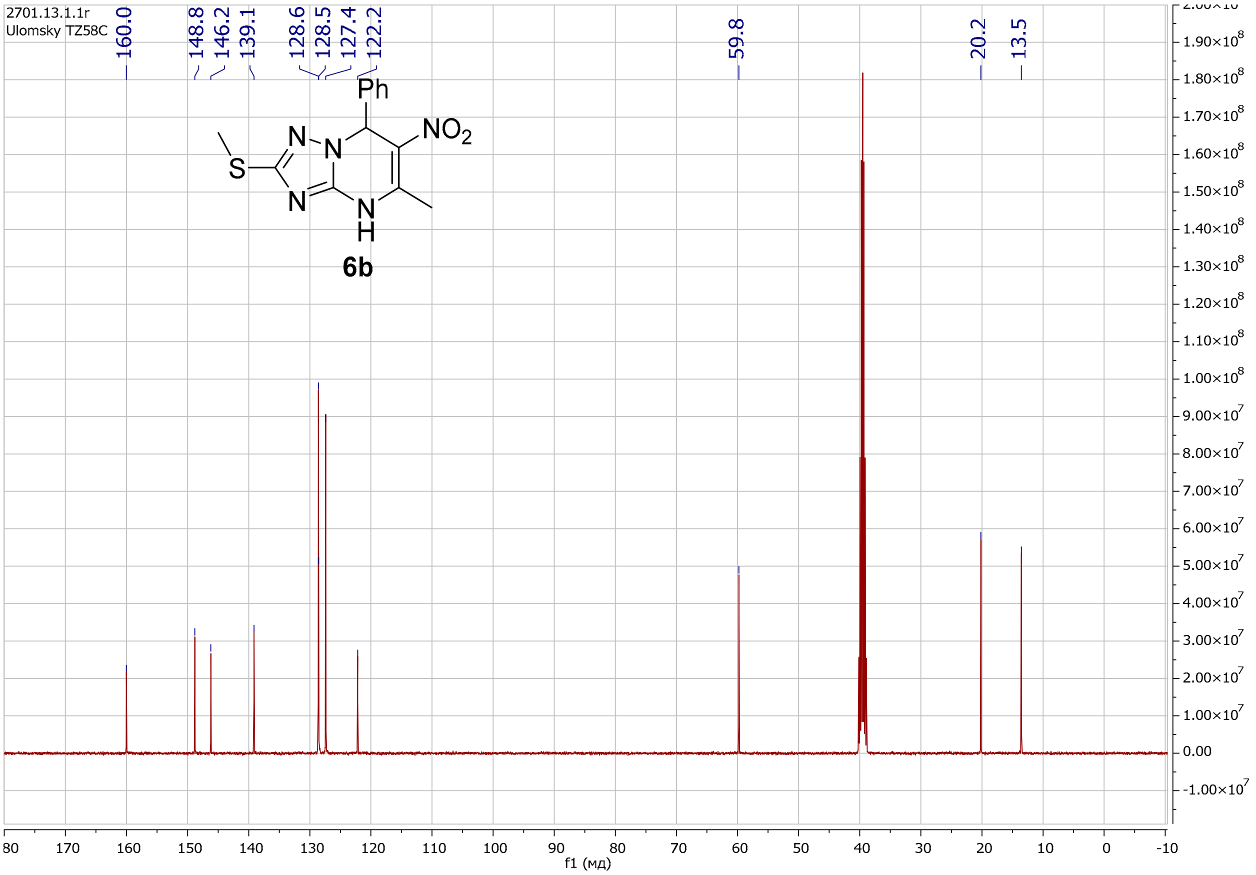
2701.13.1.1r
Ulomsky TZ58C
160.0
148.8
146.2
139.1
128.6
128.5
127.4
122.2
59.8
20.2
13.5
Ph
NO2
N
N
N
S
N
H
6b
f1 (мд)

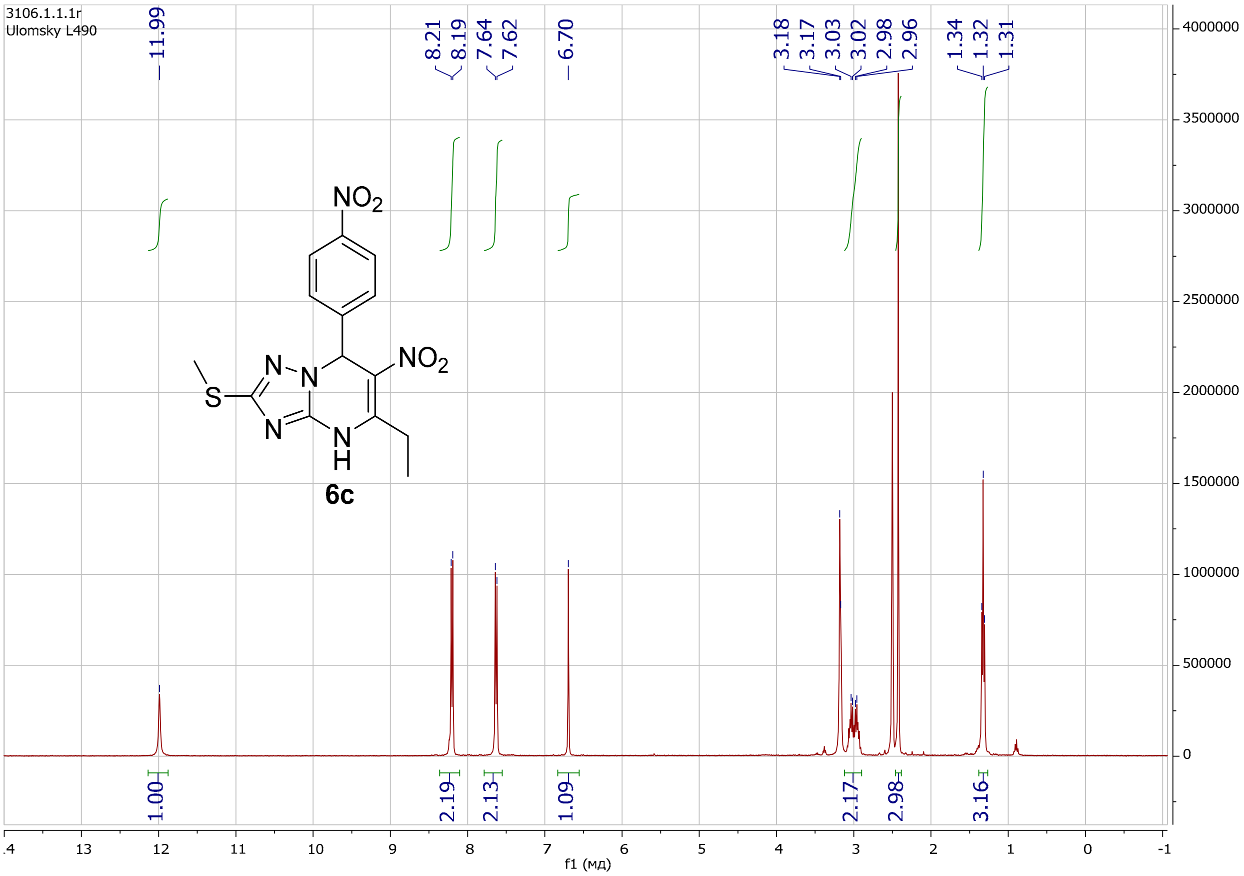
3106.1.1.1r
Ulomsky L490
11.99
8.21
8.19
7.64
7.62
6.70
3.18
3.17
3.03
3.02
2.98
2.96
1.34
1.32
1.31
1.00
2.19
2.13
1.09
2.17
2.98
3.16
NO2
NO2
N
N
N
S
N
H
6c
f1 (мд)

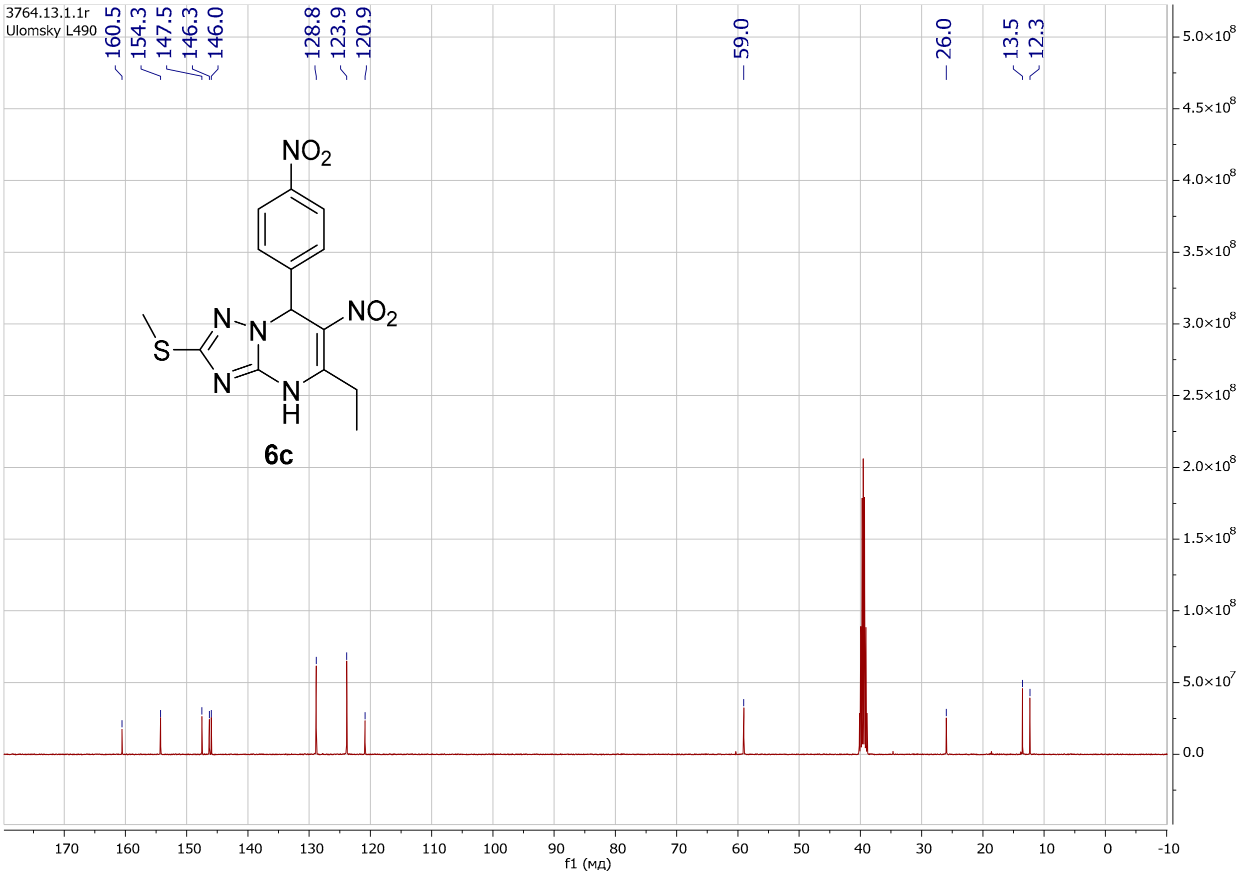
3764.13.1.1r
Ulomsky L490
160.5
154.3
147.5
146.3
146.0
128.8
123.9
120.9
59.0
26.0
13.5
12.3
NO2
NO2
N
N
N
S
N
H
6c
f1 (мд)

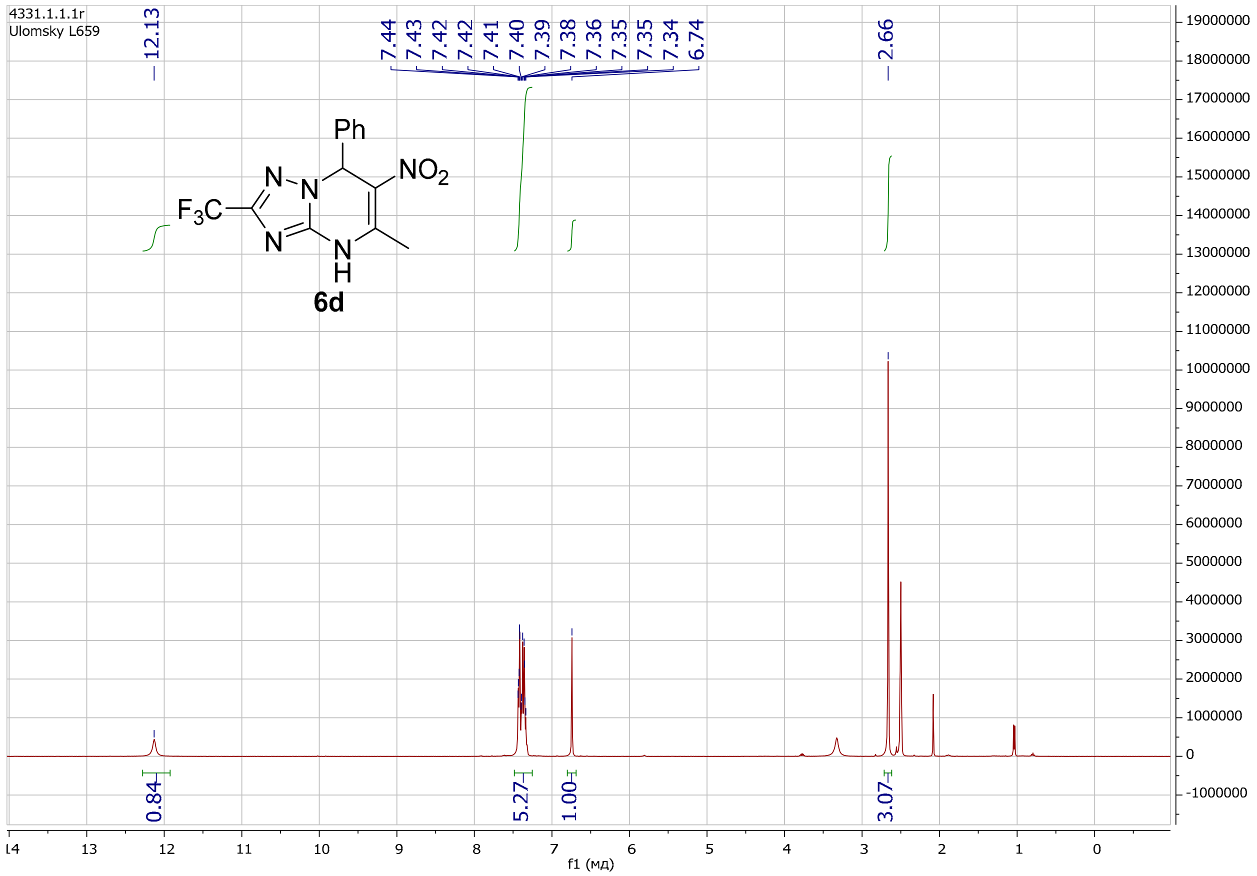
4331.1.1.1r
Ulomsky L659
12.13
7.44
7.43
7.42
7.42
7.41
7.40
7.40
7.39
7.38
7.36
7.35
7.35
7.34
6.74
2.66
Ph
$NO_2$
$F_3C$
N
N
N
N
H
6d
0.84
5.27
1.00
3.07
f1 (мд)

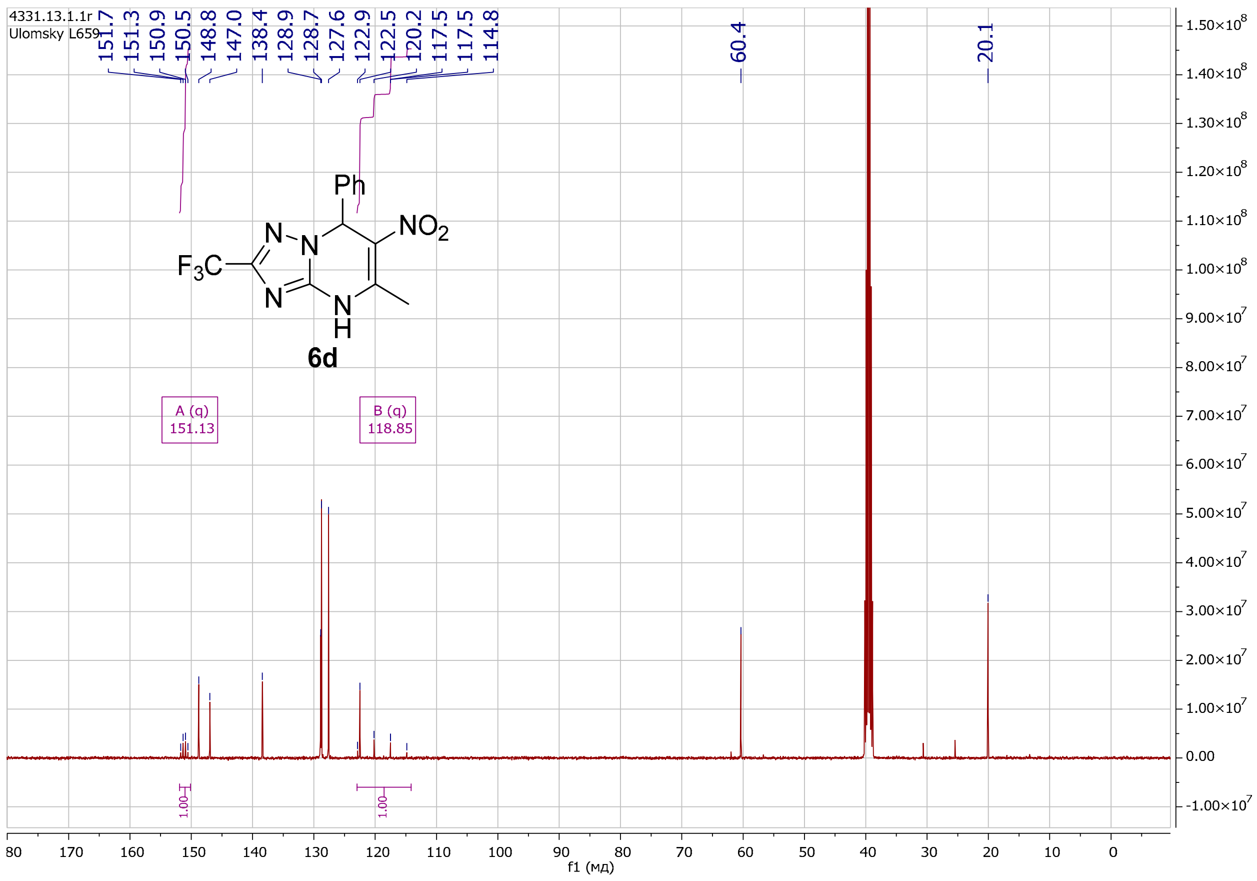
4331.13.1.1r
Ulomsky L659
151.7
151.3
150.9
150.5
148.8
147.0
138.4
128.9
128.7
127.6
122.9
122.5
120.2
117.5
117.5
114.8
60.4
20.1
Ph
$NO_2$
$F_3C$
N
N
N
N
H
6d
A (q)
151.13
B (q)
118.85
1.00
1.00
f1 (мд)

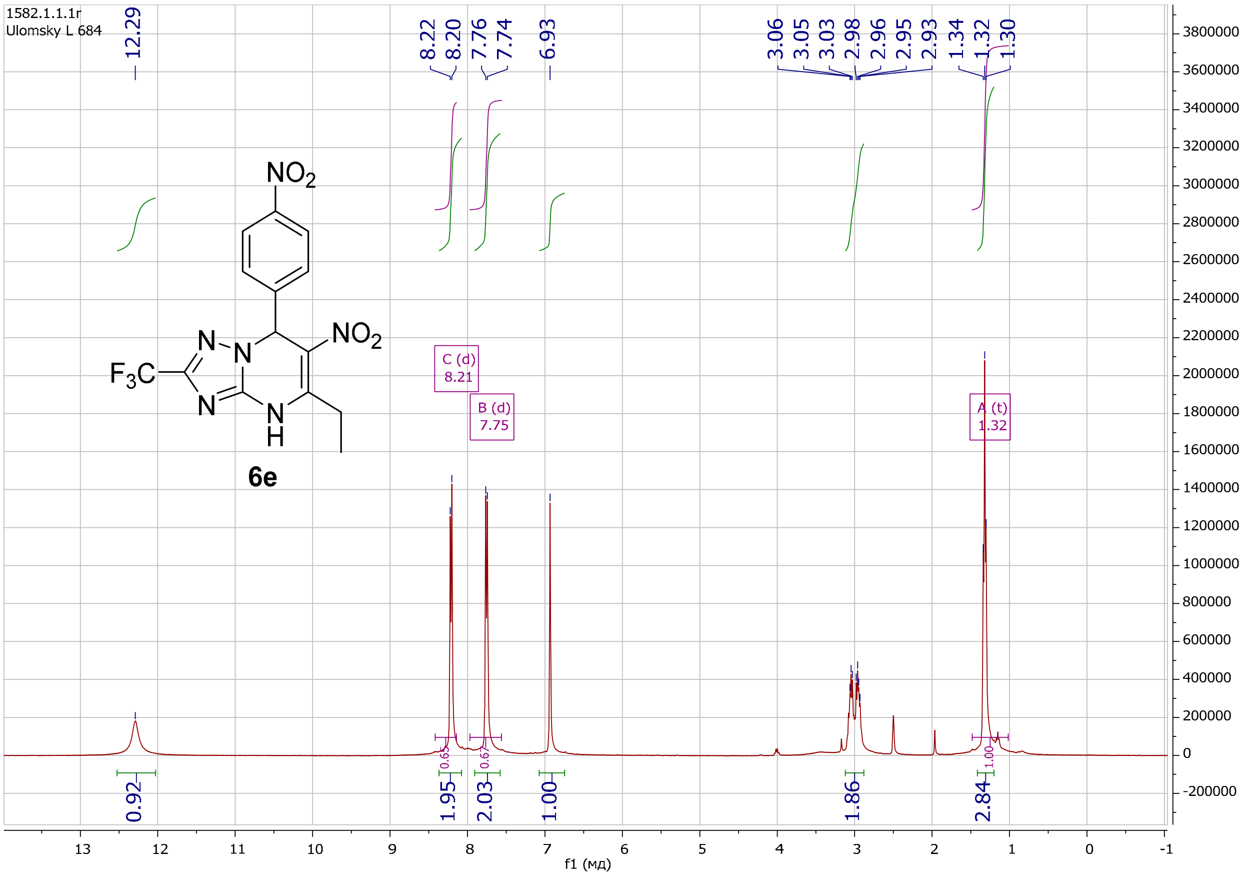
1582.1.1.1r
Ulomsky L 684
12.29
8.22
8.20
7.76
7.74
6.93
3.06
3.05
3.03
2.98
2.96
2.95
2.93
1.34
1.32
1.30
NO2
NO2
F3C
N
N
N
N
H
6e
C (d)
8.21
B (d)
7.75
A (t)
1.32
0.92
1.95
2.03
1.00
1.86
2.84
f1 (мд)

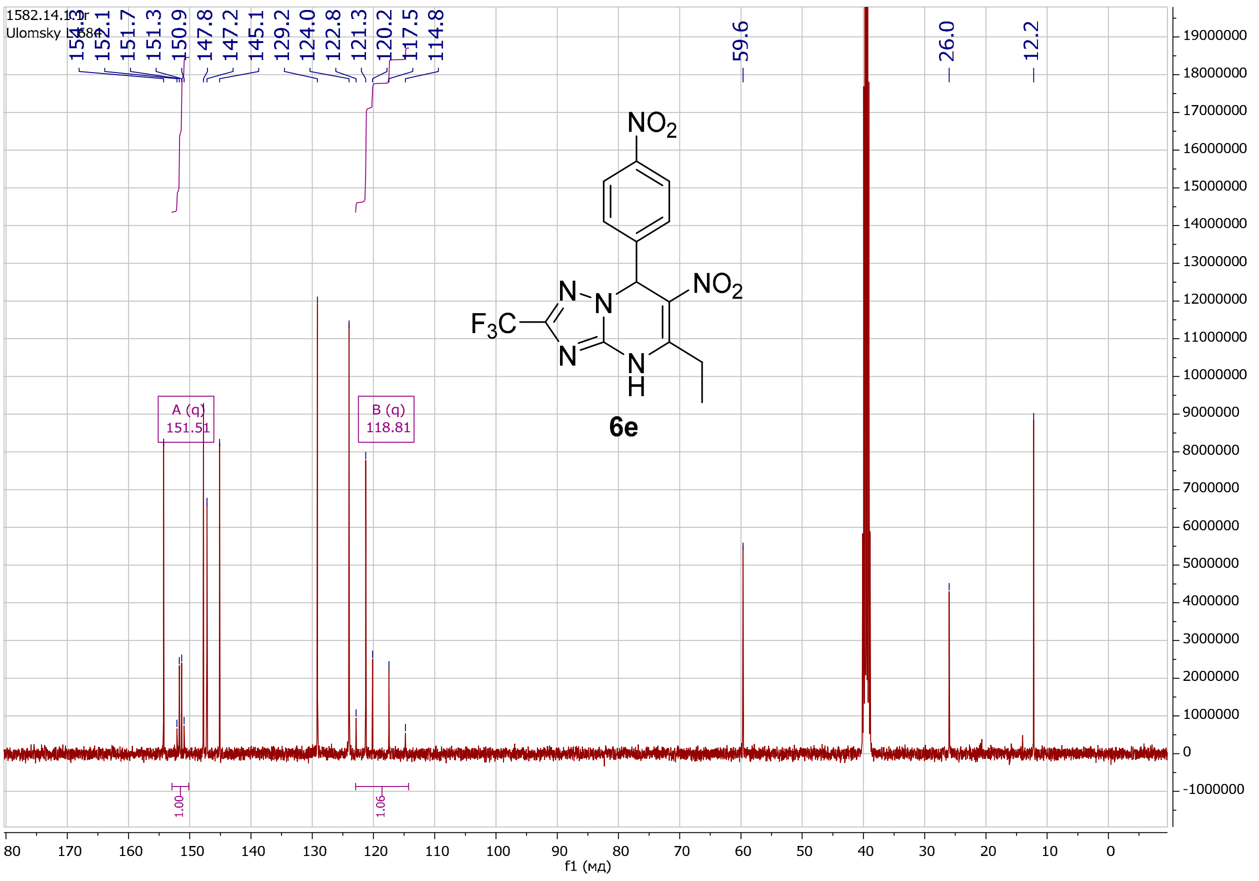
1582.14.1.1r
Ulomsky L 684
152.1
151.7
151.3
150.9
147.8
147.2
145.1
129.2
124.0
122.8
121.3
120.2
117.5
114.8
59.6
26.0
12.2
NO2
NO2
F3C
N
N
N
N
H
6e
A (q)
151.51
B (q)
118.81
1.00
1.06
f1 (мд)
